# Economical Perovskite Solar Cell Enabled by Triple Cost‐Reduction Strategies

**DOI:** 10.1002/smsc.202500451

**Published:** 2026-01-19

**Authors:** Kanokwan Choodam, Nattawut Kamjam, Noppawit Sukpan, Chaowaphat Seriwattanachai, Anuchytt Inna, KoKo Shin Thant, Ladda Srathongsian, Ratchadaporn Supruangnet, Hideki Nakajima, Anusit Kaewprajak, Pisist Kumnorkaew, Duangmanee Wongratanaphisan, Pipat Ruankham, Pasit Pakawatpanurut, Pongsakorn Kanjanaboos

**Affiliations:** ^1^ School of Materials Science and Innovation Faculty of Science Mahidol University Nakhon Pathom 73170 Thailand; ^2^ Center for Cooling and Energy‐saving Materials Faculty of Science Mahidol University Nakhon Pathom 73170 Thailand; ^3^ Synchrotron Light Research Institute 111 University Avenue Nakhon Ratchasima 30000 Thailand; ^4^ National Nanotechnology Center National Science and Technology Development Agency 114 Thailand Science Park, Phahonyothin Road Khlong Luang Pathum Thani 12120 Thailand; ^5^ Department of Physics and Materials Science Faculty of Science Chiang Mai University Chiang Mai 50200 Thailand; ^6^ Department of Chemistry, Faculty of Science Mahidol University Bangkok 10400 Thailand

**Keywords:** hole transport layer free, indoor perovskite solar cell, carbon electrode, spray coating, polyethylene glycol passivation

## Abstract

Perovskite solar cells (PSCs) are emerging as a promising technology for indoor photovoltaics due to their high efficiency and cost‐effective manufacturing. In this article, three strategies are explored to reduce costs and enable perovskite materials (PSK) as power sources for indoor internet of things (IoTs): 1) using dual perovskite absorber layer (PSK1/polyethylene glycol (PEG)/PSK2) to replace both the absorber and hole transport layers, 2) utilizing spray‐coating for perovskite deposition under ambient conditions with 45%–65% relative humidity (RH), and 3) replacing metal electrodes with carbon electrodes. The dual absorber layer improves charge transport, while the spray‐coating process minimizes solution waste, making large‐scale production more feasible. Additionally, the use of PEG as an interlayer effectively enhances defect passivation, improving charge transport and stability. The proposed carbon‐based device architecture offers the lowest material cost ($11.98 m^−2^) and the modified levelized cost of electricity for indoor light (m‐LCOE‐i) of 1.54 ¢ Wh^−1^, outperforming traditional Spiro‐OMeTAD/Au or carbon designs along with enhancing the commercial viability of PSCs. To demonstrate its practicality, connected PSCs are utilized to power IoT devices for over a month under typical laboratory lighting conditions (300–400 lux) at 40%–65% RH.

## Graphical abstract

Perovskite solar cells (PSCs) serve as cost‐effective power sources for indoor internet of things devices. The dual absorber layer improves charge transport, while the spray‐coating process minimizes solution waste and opens door for large‐scale production. Additionally, the use of polyethylene glycol as an interlayer effectively enhances defect passivation, improving charge transport and stability. The carbon‐based PSC architecture delivers competitive cost of electricity for indoor usage.

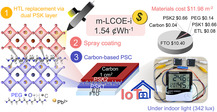

## 1 Introduction

Organic–inorganic halide perovskite materials have been used widely in solar cells, light‐emitting diodes, photodetectors, and photosensitisers in recent years.^[^ ^2^ ^]^ Perovskite materials can be modified to process many interesting properties like high broadband absorption, tunable bandgap, long charge diffusion length, and high charge carrier mobility via a low‐cost fabrication.^[^ ^1^, ^3^ ^]^ However, the commercialization of perovskite solar cells (PSCs) continues to be limited by certain challenges, such as the high cost of common hole‐transporting materials (HTMs) like Spiro‐OMeTAD, P3HT, and PTAA as well as noble metallic counter electrodes like gold and silver.^[^ ^4^, ^5^ ^]^ Conventional doped hole transport layers (HTLs) also suffer from instability, as dopants (e.g., Li‐TFSI, tBP) are hygroscopic and promote ion migration, accelerating device degradation.^[^ ^6^, ^7^, ^8^ ^]^ Efforts to replace or eliminate HTL, such as HTL‐free designs or the use of cost‐effective materials like carbon electrodes, gain traction to simplify fabrication and reduce costs.^[^ ^9^, ^10^, ^11^ ^]^ Teixeira et al. demonstrated that HTM‐free carbon‐based PSCs offer a compelling performance via spin coating process of the perovskite layer and employing a device architecture of PET/IZO/SnO_2_/Perovskite/Carbon. Although the HTM‐free design showed a slightly lower average efficiency (≈19% at 1000 lux) compared to ≈29% and ≈23% of PET/IZO/SnO_2_/Perovskite/Spiro‐OMeTAD/gold and PET/IZO/SnO_2_/Perovskite/P3HT/carbon configurations, respectively.^[^ ^12^ ^]^ Similarly, Zouhair et al. used carbon‐graphite back electrodes without an HTL to reduce cost and enhance stability, yet facing efficiency losses; introducing a 2D perovskite passivation layer significantly reduces such recombination loss, increasing open‐circuit voltage (V_OC_) and achieving up to 18.5% efficiency.^[^ ^11^ ^]^ Furthermore, Wu et al. added F_4_TCNQ to the perovskite solution, resulting in a lowered series resistance at the ITO/perovskite interface, which is attributed to the favorable interfacial band bending for more effortless hole transfer and extraction from perovskite to ITO.^[^ ^13^ ^]^

A popular spin‐coating method cannot yield commercial‐scale production due to lack of scalability and high material waste.^[^ ^14^ ^]^ To overcome such limitation, an alternative scalable technique like spray coating has been developed to enable uniform deposition over large areas.^[^ ^3^ ^]^ Chang et al. found that ultrasonic spray‐coating was a promising technique for producing the photoactive layer in PSCs. By optimizing ink formulation and drying conditions, the method achieved a power conversion efficiency (PCE) of 11.30%.^[^ ^15^ ^]^ Expanding on this approach, Huang et al. investigated the two‐step ultrasonic spray method and successfully fabricated uniform and smooth perovskite MAPbI_3_ films for efficient, large‐area solar cells. The films exhibit improved crystallinity, reduced nonradiative recombination, and enhanced charge extraction properties.^[^ ^16^ ^]^

While outdoor PSCs advance, indoor photovoltaics are being developed to power next‐generation internet of things (IoT) devices. Indoor PSCs are specifically designed to effectively harvest energy from low‐intensity light sources like LEDs and fluorescent lights.^[^ ^17^, ^18^ ^]^ Given that IoT devices often operate with extremely low power inputs (10 nW–10 μW), energy harvesting from indoor light sources has gained significant interest.^[^ ^19^ ^]^ In this context, the bandgap energy (Eg) of perovskite materials plays a crucial role in optimizing indoor performance. Srathongsian et al. achieved PCE of 31.94% with an ultralow hysteresis index by adjusting the bandgap via Cs and Br tuning and the n‐i‐p stack, which result in a convenient power source for temperature and humidity sensors under 1000 lux.^[^ ^20^ ^]^ Building upon compositional strategies for indoor photovaltaics, researchers have also investigated interfacial engineering to boost efficiency and stability in perovskite devices. Incorporating polyethylene glycol (PEG) and its derivatives into PSC structures has demonstrated significant improvements in efficiency and stability.^[^ ^21^, ^22^ ^]^

To make PSCs commercially viable, cost is the ultimate pivotal point. Our efforts aimed to overcome the barrier by combining a scalable manufacturing method with cost‐effective materials. Utilizing spray coating fabrication, hole‐free structure, and carbon electrode offer a cost‐effective trident strategy. HTL can be replaced by a low‐cost PEG interlayer along with an additional more‐p‐type perovskite layer, which helps passivate defects at the interface and improve hole transfer. Although PEG has been used as a passivation layer at electron transport layer (ETL)/PSK, PSK/HTL, and HTL/carbon interfaces,^[^ ^21^, ^22^, ^23^, ^24^, ^25^ ^]^ it has not previously been applied as an interfacial passivation layer between two different perovskite absorbers. The spray‐coated dual perovskite absorber architecture (PSK1/PEG/PSK2) sets this work apart from previous studies, offering a cost‐effective and scalable pathway toward PSC commercialization. Lastly, we demonstrated the usage of our low‐cost devices as a battery replacement by powering an indoor IoT over an extended period.

## 2 Results and Discussion

**Figure 1:**
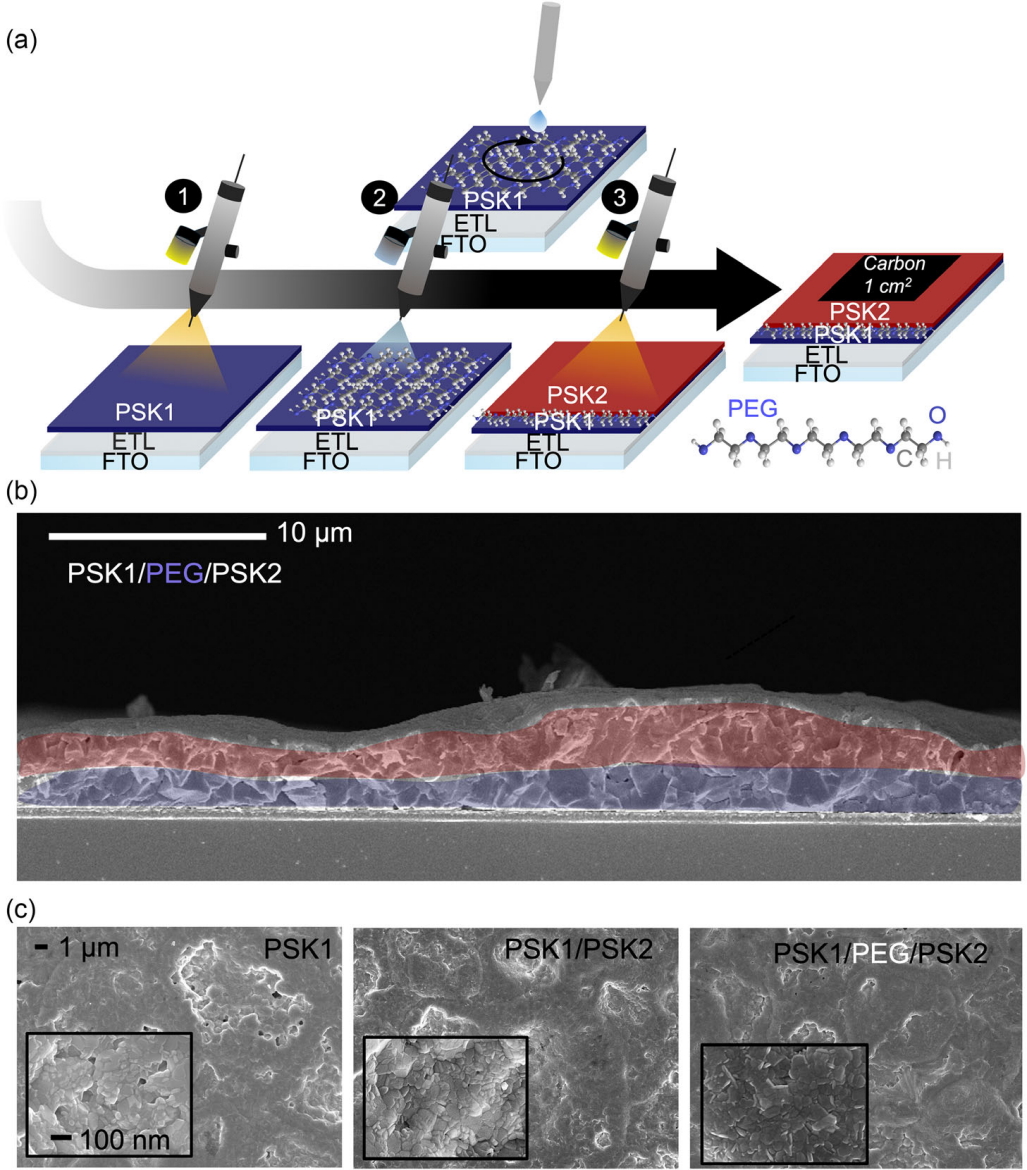
a) PSK1/PEG/PSK2 device fabrication. b) cross‐sectional SEM analysis of PSK1/PEG/PSK2 films. c) surface morphologies of PSK1, PSK1/PSK2, and PSK1/PEG/PSK2 films via SEM.

**Figure** **1**a presents the optimized planar device fabrication process using spray coating under the ambient condition with 45%–65% humidity level using an n‐i‐p device stack, Fluorine‐doped Tin Oxide glass (FTO)/Sol‐gel‐SnO_2_/SnO_2_‐nanoparticle/PSK/Carbon paste. The deposition of the perovskite film was accomplished in three main steps. First, the precursor solution of PSK1, as the bottom absorber layer, was sprayed onto a glass/FTO/Sol‐gel‐SnO_2_/SnO_2_‐nanoparticle substate heated at 130 °C on a hotplate. Generally, the first layer could be fabricated using various methods like spin coating or spray coating. However, in this work, spray coating was preferred due to its minimal solution waste and suitability for large‐scale manufacturing.^[^ ^3^, ^26^ ^]^ Likewise, a hydrophilic polymer, PEG, could be deposited by spin coating or spray coating. PEG has been widely used as a passivation layer for PSCs, as shown in **Table** **1**. It has been utilized as passivation layers at different interfaces like ETL/PSK, PSK/HTL, and HTL/carbon. However, it had not yet been applied to passivate between different types of PSK absorbers. The table indicates that PEG can enhance PSCs by acting as a Lewis base, where the —C=O groups of PEG effectively passivate surface defects in the perovskite thin film through interactions with Pb^2+^ ions.^[^ ^22^ ^]^ Additionally, hydrogen‐bonding interactions between the PEG‐PCBM buffer layer and the perovskite layer further stabilize the ETL/perovskite interface.^[^ ^21^ ^]^ PEG treatment also improves the morphology and conductivity of the PEDOT chain packing.^[^ ^27^ ^]^ On the HTL side, PEG acts as a bridge molecule, optimizing the work function for better energy level alignment while passivating defects and promoting perovskite crystallization.^[^ ^23^ ^]^ Furthermore, PEG can form self‐supporting composites within the carbon paste, enhancing the conductivity of carbon films and improving electrode performance.^[^ ^28^ ^]^ By reducing traps and defects at the perovskite/CuSCN interface, PEG lowers the potential barrier, and therefore, enhances hole extraction.^[^ ^29^ ^]^ Finally, a PEG‐passivated carbon electrode via soaking carbon paste in a PEG/ethanol solution, drying, and hot‐pressing onto the perovskite layer strengthens the interfacial carrier transport and bonding.^[^ ^30^ ^]^ Overall, PEG serves as an effective passivation layer, reducing defect density and facilitating efficient charge transfer. For the third step, PSK2 was sprayed as the top absorber/HTL layer. The third step was only possible through spray coating, as the solvent used could dissolve PSK1 if performed by other processes.^[^ ^31^, ^32^ ^]^

**Table 1:** Summary of device structures and mechanisms in PSCs with PEG passivation.

Interface	Device structure	Mechanism	Reference
HTL/PSK	ITO/PTAA/PEGDA/MA PbI_2.55_ Br_0.45_/PC_61_BM/Al 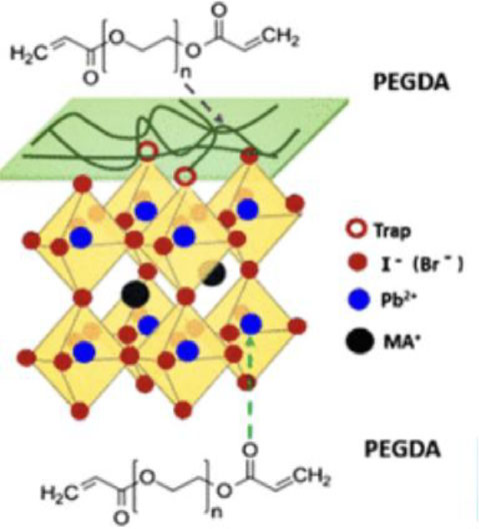	a. PEG acts as a Lewis base and passivates undercoordinated Pb^2+^ ions.	[22]
ETL/PSK	ITO/SnO_2_/PCBM‐PEG/ MAPbI_3_/Ag 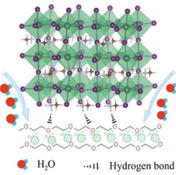	a. Hydrogen‐bonding interactions between the PEG‐PCBM buffer layer and the PSK layer stabilize interface and reduce charge recombination.	[21]
HTL/PSK	ITO/PEDOT:PSS/PEG‐200/MAPbI_3_/PC_60_BM/Ag 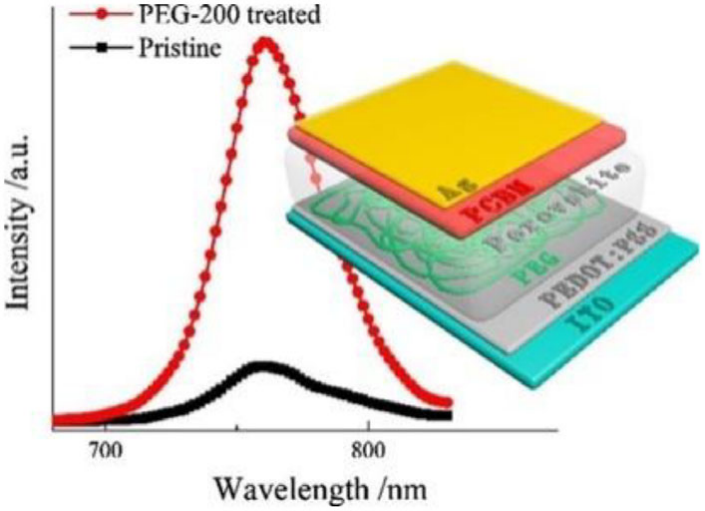	a. PEG treatment enhances the morphology and conductivity of the PEDOT layer.	[27]
HTL/PSK	ITO/PEDOT:PSS /PEG/(FASnI_3_)_0.6_(MAPbI_3_)_0.4_/C_60_/BCP/Ag 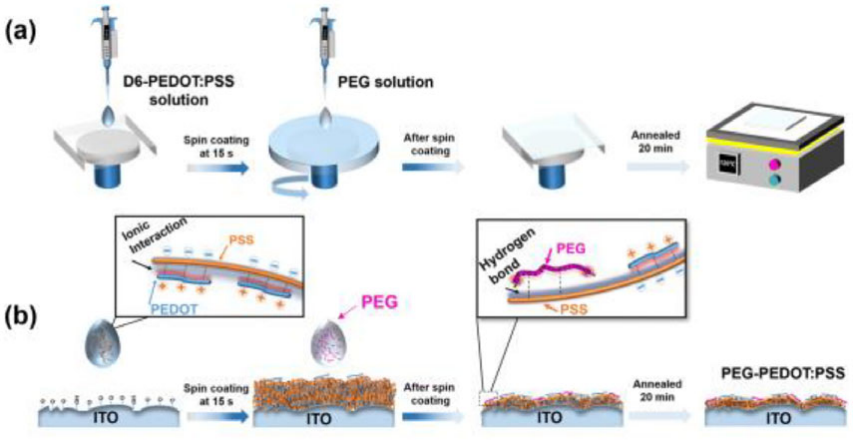	a. PEG functions as a bridge molecule at the HTL side. b. PEG optimizes the work function, enabling better energy level alignment between the HTL and the PSK layer.c. PEG passivates interfacial defects and promotes perovskite crystallization.	[23]
HTL/carbon	FTO/TiO_2_/Cs_0.17_FA_0.83_Pb(I_0.83_Br_0.17_)_3_/Spiro‐OMeTAD/Carbon 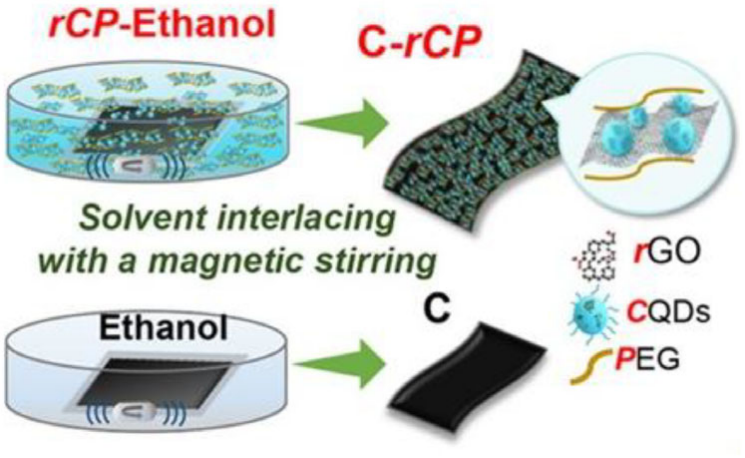	a. PEG forms self‐supporting composites within the carbon paste, leading to higher conductivity.	[28]
PSK/HTL	ITO/SnO_2_CoCl_2_/(MAFA)Pb(IBr)_3_/PEG/CuSCN/Au 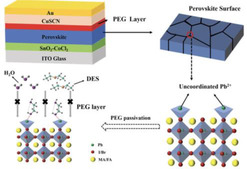	a. PEG reduces traps and defects at the perovskite/CuSCN interface, lowering the interfacial potential barrier and enhancing hole extraction.	[29]
PSK/Carbon	ITO/TiO_2_/Perovskite/PEG/Carbon 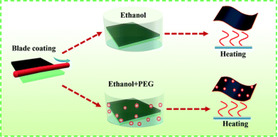	a. Soaking the carbon paste in a PEG/ethanol solution followed by drying and hot‐pressing results in enhanced interfacial bonding carrier transport.	[30]
PSK/Carbon	ITO/SnO_2_/CsPbI_2_Br/PEG/Carbon 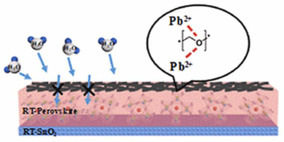	a. PEG, as an interface passivation layer, effectively reduces the defect density in the PSK layer and promotes efficient charge transfer from PSK to the carbon electrode.	[64]
PSK1/PSK2	FTO/Bilayer‐SnO_2_/PSK1/PEG/PSK2/Carbon 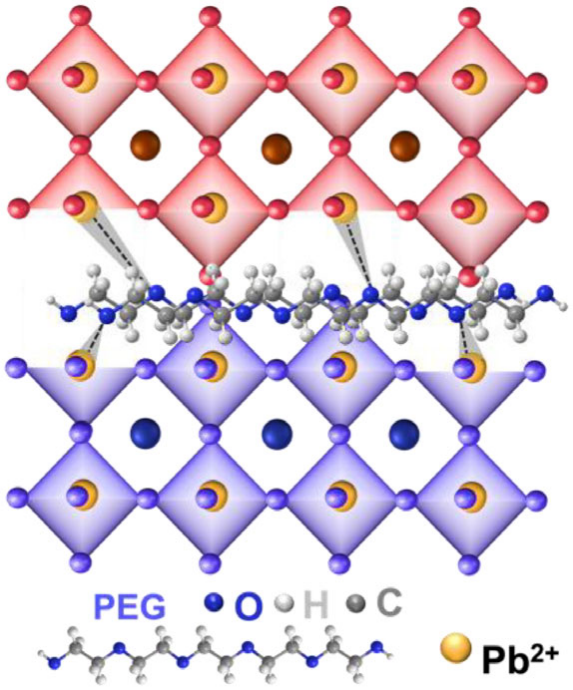	a. PEG is utilized as an interlayer between two perovskite absorbers to effectively passivate defects.b. PEG improves device performance by enhancing charge transport.	This work

A critical issue in hole‐free PSCs arises from the unwanted recombination of electrons and holes.^[^ ^5^ ^]^ The addition of PSK2 helps directing the flow of charge toward a designed direction.^[^ ^32^ ^]^ Additionally, phenethylammonium iodide (PEAI) was included as an additive in PSK2 to passivate defects by filling iodide vacancies to realize excellent long‐term stability.^[^ ^33^, ^34^, ^35^, ^36^ ^]^ The addition of PEAI is also shown to enhance the electron‐blocking ability from previous literatures.^[^ ^33^, ^34^, ^36^ ^]^ The specific absorber composition (PSK1/PEG/PSK2) was selected based on band alignment, as illustrated in Figure S1, Supporting Information. PSK1 exhibits a stronger n‐type character^[^ ^37^, ^38^ ^]^ than that of PSK2;the result guided the design to optimize charge transport and overall device performance.

All three types of absorber stacks including perovskite (PSK1), two types of perovskites (PSK1/PSK2), and two types of perovskites with a passivation layer (PSK1/PEG/PSK2) were controlled to processes similar thickness values by adjusting flow rate and spray head speed.^[^ ^3^ ^]^ The cross‐sectional SEM image of (PSK1/PEG/PSK2) in Figure 1b, with the control shown in Figure S2, Supporting Information, shows that the thicknesses from spraying coating could vary between 2 and 4 μm according to a droplet pattern. Usually, spray coating results in high surface roughness and a droplet‐like pattern, and when the film is too thin, it can lead to short‐circuiting.^[^ ^39^, ^40^ ^]^ Moreover, appropriately thicker perovskite films are commonly used in HTL‐free solar cells to maximize the absorption of incident light, which is critical for achieving high photocurrents.^[^ ^41^ ^]^ Surface morphologies of PSK1, PSK1/PSK2, and PSK1/PEG/PSK2 are shown in Figure 1c at 50k and 3k magnifications, illustrating no noticeable differences. PSK1 exhibits more pinholes compared to those of PSK1/PSK2 and PSK1/PEG/PSK2, in an agreement with the results from an optical microscope in Figure S3, Supporting Information at a 10x magnification. When light was applied from the bottom side, more pinholes became visible.

Atomic force microscope (AFM) results showed that the roughness of glass/PSK2 was 553 nm, which is higher than 439 nm of glass/PSK1 layer as shown in Figure S4, Supporting Information. Previously observed in large‐scale processes like spray coating and slot‐die printing, increased roughness has tendency to improve the interface between PSK and HTL/carbon, allowing for more efficient charge separation and extraction, as more surface areas are available for contact, thereby reducing the resistance at the interface.^[^ ^42^, ^43^ ^]^ The rough surface also reduces the likelihood of pinhole formation, which is often caused by incomplete film coverage or gaps between grains.^[^ ^44^, ^45^ ^]^

Moreover, comparison with previous reports using similar perovskite absorber structures (Table S1, Supporting Information) shows that, in conventional architectures (ETL/PSK/HTL), graded energy band structures are commonly employed to enhance charge transport.^[^ ^31^, ^32^ ^]^ For instance, previous works introduced 2D/3D perovskite heterojunctions via interfacial ion exchange, where the 2D capping layer passivates defects, enhances hydrophobicity, and forms a graded band structure for improved charge extraction.^[^ ^33^ ^]^ In HTL‐free devices, 2D perovskite passivation layers have been used as electron‐blocking layers.^[^ ^11^ ^]^ Wide bandgap perovskite materials have been incorporated into 3D/3D+Br stacking structures to improve the perovskite/carbon electrode interface.^[^ ^46^ ^]^ In this work, we demonstrate a dual perovskite absorber architecture (PSK1/PEG/PSK2) that replaces both the traditional absorber and HTL.

**Figure 2:**
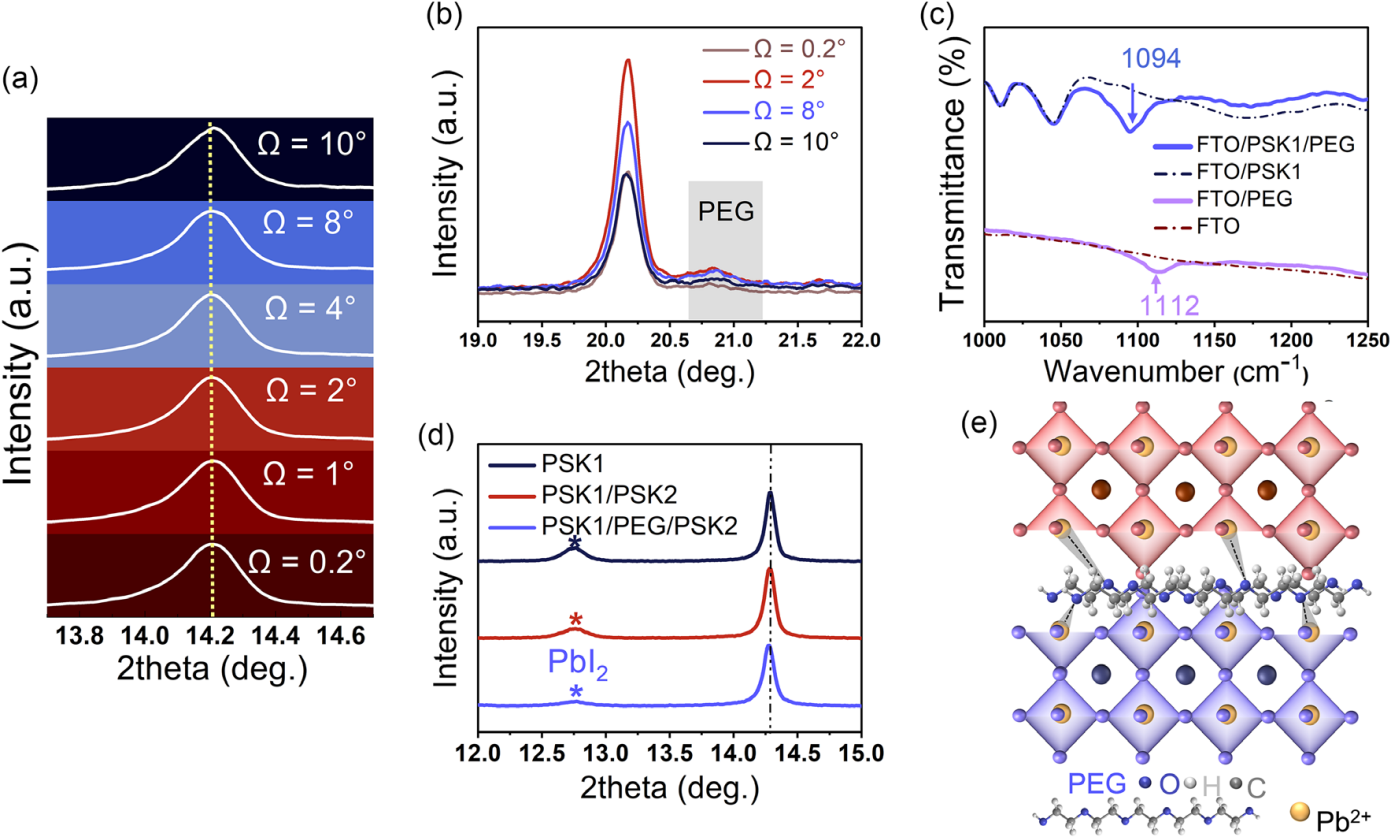
a) Diffraction profiles for the <110> plane of a FTO/ETL/PSK1/PEG/PSK2 film identified by GI‐XRD at different grazing angles; higher angles present signals from buried interface toward the FTO substate. The red‐to‐blue gradiences represent PSK formations from PSK2/air to ETL/PSK1 interfaces. b) PEG‐6000 peaks in the PSK1/PEG/PSK2 film at different grazing angles. c) FTIR analysis of PEG interaction with uncoordinated Pb^2+^ within the PSK system. d) X‐ray diffraction analyses of various fabrication stacks of interest. e) PEG passivation schematic: the interaction between C—O—C and uncoordinated Pb^2+^.

To confirm the dual perovskite architecture, grazing incidence X‐ray diffraction (GI‐XRD) was performed on the PSK1/PEG/PSK2 film. As shown in **Figure** **2**a, the diffraction profile at a 10° grazing angle reveals the peak signal of PSK1 crystals compared to the diffraction profile at a 0.2° grazing angle which represents the PSK2 crystals. Evidently, the increase in the grazing angle is accompanied by a shift in the diffraction peak of the (110) perovskite crystal plane toward smaller angles.^[^ ^32^ ^]^ This movement of the diffraction peak can be attributed to the increase in lattice spacing caused by PEAI addition to form the PSK2 material.^[^ ^47^, ^48^ ^]^ As expected, at high grazing angles like 8° and 10°, the FTO plane can be observed as shown in Figure S5, Supporting Information. Typically, a PEG peak can appear over a wide range of 2*θ* from 19° to 28°, indicating its amorphous nature.^[^ ^49^, ^50^, ^51^ ^]^ As shown in Figure 2b, for our case, a distinct peak is observed at 2*θ* = 20.8°. A strong peak of FTO/PEG is observed at PEG is observed with the highest intensity at the grazing angle of 2°, further confirming its position at the PSK1/PSK2 interface. The Fourier transform infrared (FTIR) results in Figure 2c, the C—O stretching vibration peak shifts from 1112 cm^−1^ to a lower wavenumber located at 1094 cm^−1^, which suggests the interaction between PEG and perovskite.^[^ ^52^, ^53^ ^]^ The XRD patterns of PSK1, PSK1/PSK2, and PSK1/PEG/PSK2, shown in Figure 2d, suggest the presence of inactive PbI_2_ crystals at a 2*θ* value of 12.6°. With PEG passivation via the C—O—C bonds of PEG and uncoordinated Pb^2+^ as illustrated in Figure 2e, the inactive perovskite phase is diminished,^[^ ^21^, ^22^, ^23^, ^29^ ^]^ while the intensities of the active perovskite phases remain unchanged.

**Figure 3:**
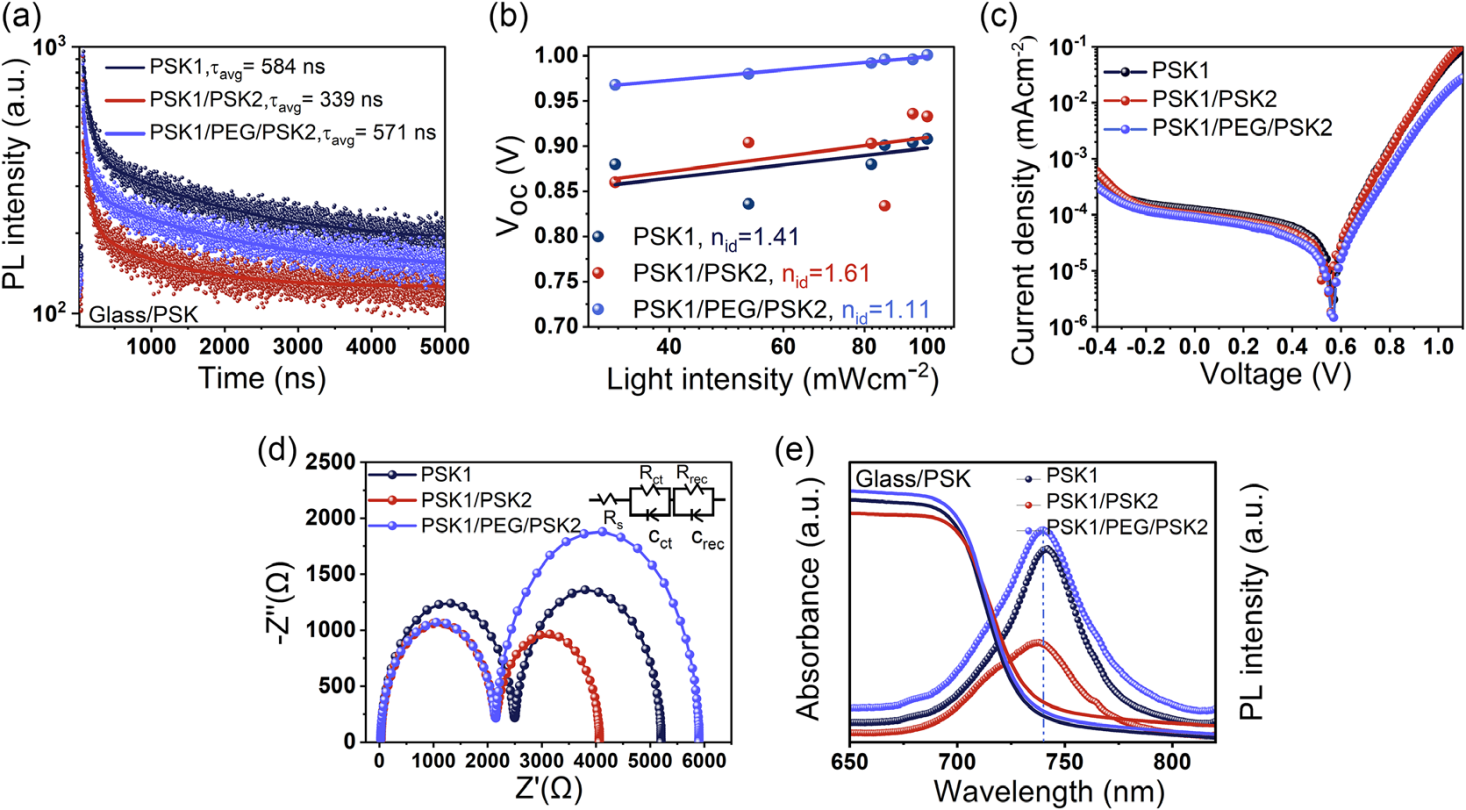
a) Light intensity versus V_oc_ performance of PSK1, PSK1/PSK2, and PSK1/PEG/PSK2 devices to identify ideality factors. b) TRPL decays of glass/PSK1, glass/PSK1/PSK2, and glass/PSK1/PEG/PSK2 films, illustrating differences in carrier recombination lifetimes. c) Dark J–V characteristics of PSCs based on PSK1, PSK1/PSK2, and PSK1/PEG/PSK2 structures, showing variations in reverse leakage currents. d) Nyquist plots from EIS of PSK1, PSK1/PSK2, and PSK1/PEG/PSK2 devices, providing insights into charge transport and recombination. e) Absorption and PL spectra of glass/PSK1, glass/PSK1/PSK2, and glass/PSK1/PEG/PSK2 films, demonstrating the effect of PEG‐induced defect passivation without affecting light absorption.

V_oc_‐light intensity dependance measurements were performed as illustrated in **Figure** **3**a. According to the equation, $V_{oc}\approx n_{id}KTln\left(L\right)/q$, the ideality factor (*n_id_*) is associated with the recombination mechanism; *n_id_* = 1 indicates band‐to‐band recombination, whereas *n_id_* = 2 corresponds to trap‐assisted recombination or Shockley–Read–Hall (SRH) recombination.^[^ ^20^ ^]^ In terms of the calculated *n*_id_, PSK1/PEG/PSK2 has the lowest *n_id_* of 1.11, suggesting predominant band‐to‐band recombination with less SRH recombination. In contrast, PSK1 and PSK1/PSK2 systems have n_id_ values of 1.41 and 1.61, respectively; such values closer to 2 indicate higher trap‐assisted recombination.^[^ ^24^ ^]^ The use of PEG assists in altering defect‐dominated carrier recombination to favorable band‐to‐band counterpart.^[^ ^54^, ^55^, ^56^ ^]^

The charge carrier lifetimes of the perovskite films are shown in Figure 3b. The parameters were determined by biexponential fitting, described by the equation:I(t)=A_1_exp(−t/t_1_) + A_2_exp(−t/t_2_), where A_1_ represents the contribution of the fast decay component (t_1_), which is associated with nonradiative recombination processes such as defects at interfaces or surfaces. A_2_ corresponds to the contribution of the slow decay component (t_2_), which is related to intrinsic band‐to‐band radiative recombination within the bulk material.^[^ ^20^ ^]^ PSK1, PSK1/PSK2, and PSK1/PEG/PSK2 exhibit average lifetimes (t_avg_) of 584, 339, and 571 ns, respectively. The time‐resolved photoluminescence (TRPL) fitting parameters in Table S2, Supporting Information, reveals that the incorporation of PEG significantly improves the recombination dynamics, causing PSK1/PEG/PSK2 to have comparable A and t values to those of PSK1 with one less interface, as PEG effectively harmonizes two distinct perovskite absorbers as previously demonstrated for ETL/PSK and PSK/HTL interfaces.^[^ ^54^, ^55^, ^57^, ^58^ ^]^ On contrary, PSK1/PSK2 processes large t_avg_ from nonradiative recombination at the nonpassivated surface.

The analysis of the dark J–V curves, as shown in Figure 3c, indicates that a lower leakage current corresponds to more effective blocking of reverse carriers and fewer interfacial or bulk defects.^[^ ^59^ ^]^ The PSK1, PSK1/PSK2, and PSK1/PEG/PSK2 devices exhibit reverse leakage current densities of 4.04 × 10^−6^, 1.85 × 10^−6^, and 1.71 × 10^−6^ mA cm^−2^ at 0.56 V, respectively. The lowest reverse leakage current observed in the PSK1/PEG/PSK2 device suggests that carrier transport and collection are enhanced by the incorporation of the PEG layer, consistent with the trend observed in the photoluminescence (PL) lifetime results.^[^ ^24^, ^60^ ^]^

In Figure 3d, the Nyquist plots exhibit two main regions, with the equivalent circuit for charge transfer and recombination processes.^[^ ^61^ ^]^ Reductions in charge transfer resistance (R_ct_) for the PSK1/PSK2 and PSK1/PEG/PSK2 devices are attributed to better charge quenching from having two perovskite absorbers. Furthermore, the higher charge recombination resistance (R_rec_) observed in the PSK1/PEG/PSK2 device links to reduced recombination losses.^[^ ^62^, ^63^ ^]^ Hence, the incorporation of PEG suppresses carrier recombination at the PSK interface, making seamless connection for charge transfer similar to that of homogenous PSK1, in agreement with above TRPL results.^[^ ^64^, ^65^ ^]^

In Figure 3e, the absorption spectra of all films are nearly identical, indicating that the PEG layer does not significantly impact light absorption in the visible wavelength range. As expected, PL intensities are considerably higher for PSK1/PEG/PSK2 and PSK1 films compared to that of PSK1/PSK2.^[^ ^66^, ^67^ ^]^

**Figure 4:**
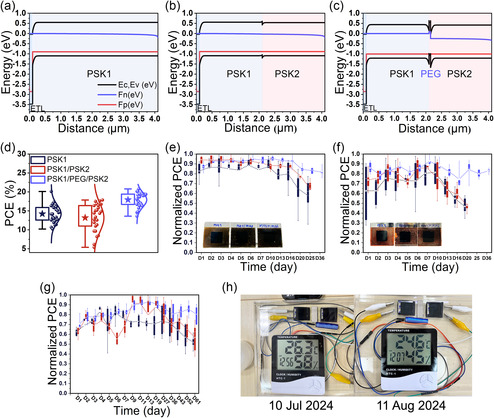
a) Energy band diagrams of PSK1, b) PSK1/PSK2, and c) PSK1/PEG/PSK2 structures simulated using SCAPS. d) Device performance of PSK1, PSK1/PSK2, and PSK1/PEG/PSK2 under 1000 lux indoor illumination. e) Thermal stability of unencapsulated devices at 65 °C in ambient conditions. f) Light stability of unencapsulated devices under low‐intensity white light (8 h/day) in ambient conditions. g) Stability of unencapsulated devices stored in a humidity‐controlled cabinet (40%–60% RH) in the dark. h) Performance demonstration of a PSK1/PEG/PSK2 device powering a thermo‐hygrometer (HTC‐1 model) under low light.

Table S3, Supporting Information, compares various photovoltaic simulation tools and indicates that, in terms of cost, open‐source availability, and usability, solar cell capacitance simulator (SCAPS) is selected for this work due to its intuitive interface and minimal setup, making it particularly suitable for rapid simulations of thin‐film and other photovoltaic devices. Moreover, SCAPS is highly valued for its customizable configurations, access to internal variables, and ability to be calibrated with experimental results.^[^ ^68^, ^69^, ^70^ ^]^ SCAPS‐1D was used to investigate single‐ and dual‐layer perovskite structure with and without PEG, focusing on the role of PEG as an interfacial layer. The work function obtained from ultraviolet photoelectron spectroscopy (UPS) is combined with the bandgap from ultraviolet–visible spectrophotometry (UV–Vis) to construct the energy bands of PSK1, PSK1/PEG, and PSK2, as shown in Figure S6, Supporting Information. The electron affinity (conduction band), thickness, bandgap, and other parameters listed in Table S4, Supporting Information, were incorporated to simulate the band bending of the PSK1 structure (**Figure** **4**a), the PSK1/PSK2 structure (Figure 4b), and the PSK1/PEG/PSK2 structure (Figure 4c). The conduction band offset (CBO) values for the PSK1, PSK1/PSK2, and PSK1/PEG/PSK2 structures were 0.00, −0.18, and +0.49 eV,^[^ ^54^ ^]^ respectively, as shown in Table S5, Supporting Information, a negative CBO creates a “cliff” structure that facilitates electron transfer but may increase interfacial recombination. In contrast, a positive CBO forms a “spike” that effectively blocks charge transport.^[^ ^71^, ^72^ ^]^ The addition of a thin PEG layer introduces a spike at the interface, forming a barrier to photogenerated charge carrier flow toward the electrode.^[^ ^73^, ^74^ ^]^ Severe current limitation caused by a large spike can be associated with excessively thick PEG layers, resulting in distortions of the current–voltage curves and reduced fill factor (FF).^[^ ^75^ ^]^ For the thin PEG in our case, the PSK1/PEG/PSK2 structure still shows a slightly lower FF compared to those of PSK1 and PSK1/PSK2 devices. However, as shown in Figure S7, Supporting Information, the simulated device performance of PSK1/PEG/PSK2 does not change significantly. In agreement with the simulated open‐circuit voltage (V_oc_) values, previous studies report that a spike can lead to charge accumulation at the interface, generating excess electrostatic potential, which enhances V_oc_.^[^ ^71^ ^]^ Furthermore, electrons can tunnel through a thin layer of PEG.^[^ ^76^ ^]^ To identify appropriate PEG thicknesses experimentally, concentration (0.75, 1.50, and 3.00 mg in 1 mL of CB) and molecular weight (6k and 20k) optimizations are shown in Figure S8, Supporting Information. The use of 1.50 mg of 6k PEG results in best performance. Excessive PEG concentration or molecular weight brings about defects along with an insulating property, reducing current density (J_sc_), FF, and PCE.^[^ ^77^, ^78^ ^]^

For the experimental results, indoor light performance was evaluated under 1000 lux LED illumination. As shown in Figure S9, Supporting Information, the spectrum of the 6500 K LED light source in the testing box was directly measured using a spectroradiometer equipped with a certified EOP‐146 detector. The incorporation of PEG as a passivation layer mitigates defects at the PSK1/PSK2 interface, resulting in an average J_sc_ of 120 μA cm^−2^ for the PSK1/PEG/PSK2 device, compared to 108 μA cm^−2^ for both the PSK1 and PSK1/PSK2 control devices. Additionally, the average V_oc_ and FF of the PSK1/PEG/PSK2 devices are higher than those of the control devices, as shown in Figure S10, Supporting Information. Additionally, the optimized PSK1/PEG/PSK2 device exhibits the highest R_sh_ and lowest R_s_, confirming that PEG effectively passivates defects and improves interfacial contact,^[^ ^79^ ^]^ thereby reducing leakage currents, enhancing charge transport, and contributing to the improved V_oc_ and FF.^[^ ^80^, ^81^ ^]^

Figure 4d, the champion PCE value of the PSK1/PEG/PSK2 devices is around 21% with an average of 18% across 25 cells, representing a significant improvement over the control devices. The PSK1 devices achieve a maximum PCE of 20% (average at 14%), while the PSK1/PSK2 devices reach a maximum of 18% (average at 13%). Additionally, the device performance of the PSK1/PEG/PSK1 structure is close to that of PSK1, suggesting the important contribution of the graded band structure in PSK1/PEG/PSK2, as shown in Figure S11, Supporting Information.

The normalized performances over time for the PSK1, PSK1/PSK2, and PSK1/PEG/PSK2 devices, all of which were unencapsulated, are evaluated under various conditions following modified international summit on organic solar cell stability (ISOS) protocols.^[^ ^82^ ^]^ In Figure 4e, the devices were tested under heat at 65 °C in ambient conditions, and Figure 4f shows the device performance under white light illumination with light cycling: 8 h under 1000 lux and 16 h in dark per day in ambient conditions. Under both heat and light exposure, the PSK1/PEG/PSK2 devices maintain more than 80% of its initial performance for over 36 days. In contrast, PSK1 and PSK1/PSK2 devices drop below 80% after 20 days under heat conditions and after 10 days under light exposure. In Figure 4g, the devices were tested in the dark and stored in a humidity‐controlled cabinet with 40%–60% relative humidity (RH). The performance of the PSK1/PEG/PSK2 devices remains above 80% for over 61 days, while PSK1 and PSK1/PSK2 devices drop below 80% after 27 days. The PSK1/PEG/PSK2 configuration demonstrates enhanced stability under a variety of environmental challenges. High temperature or illumination accelerates ion migration (such as I^−^, MA^+^, or vacancies) within the lattice, leading to perovskite degradation.^[^ ^83^, ^84^, ^85^ ^]^ Under these conditions, the PEG layer in the PSK1/PEG/PSK2 structure effectively passivates defects and deaccelerate ion movements, resulting in significantly improved stability.

To demonstrate the aptitude of PSK1/PEG/PSK2 structure for powering low‐power IoTs devices under normal indoor light ambience, two such devices connected in series were placed on the bench in our laboratory (342 lux) as shown in Figure S12, Supporting Information, and could easily power a thermo‐hygrometer (HTC‐1 model) continuously over 32 days, enabling the measurement of RH, temperature, and time, as shown in Figure 4h. Their power output was recorded (Table S6, Supporting Information). The connected device could generate a sufficient voltage to operate IoT devices, which require 1.5 V. The solar cell generates 0.0896 mW, which is more than enough to power the sensor system, which requires 0.0275 mW.

**Figure 5:**
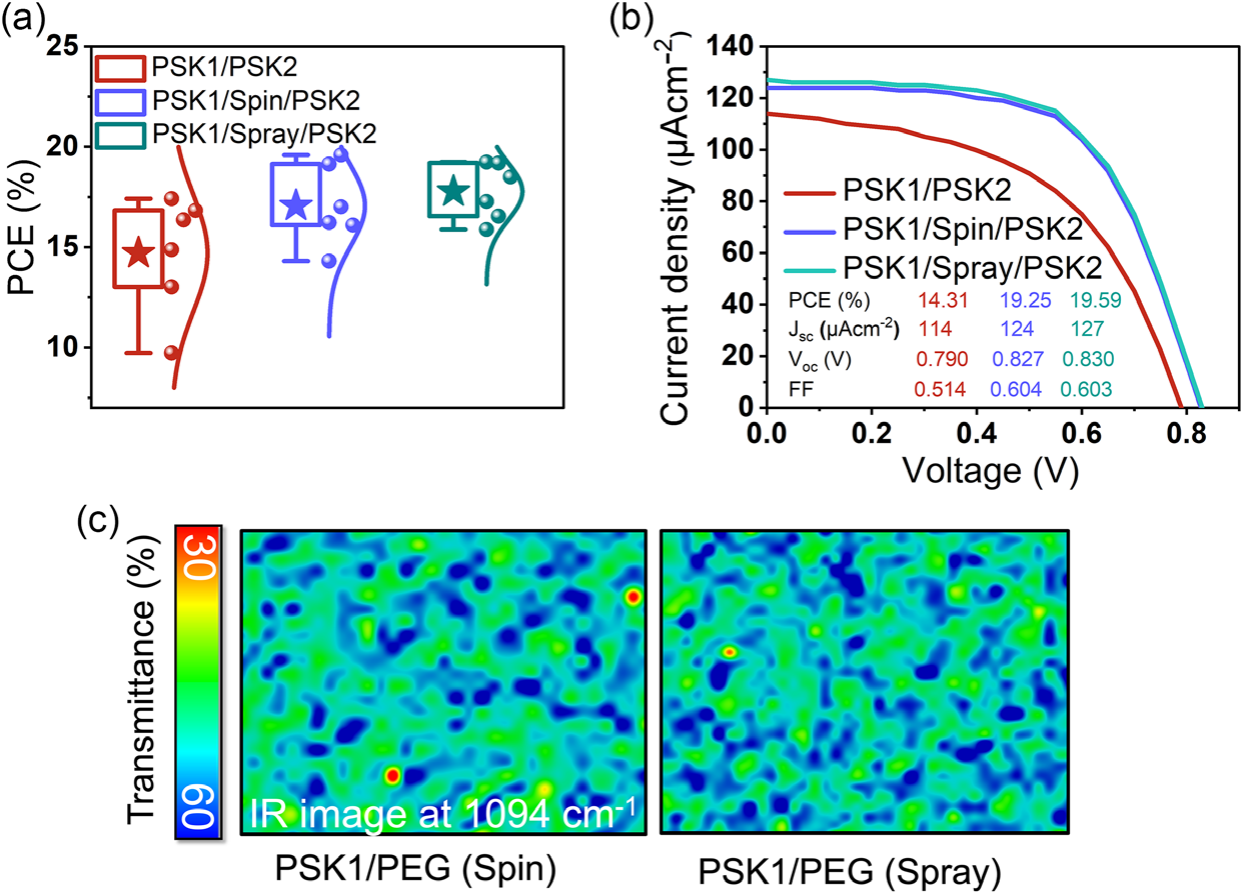
a) Device performance comparison, and b) J–V measurements under 1000 lux LED for PSK1/PSK2, PSK1/spin‐coated PEG/PSK2, and PSK1/spray‐coated PEG/PSK2 structures. c) FTIR mapping of PEG coverage at 1094 cm^−1^ on spin‐ and spray‐coated perovskite films.

The PSK/PEG/PSK2 structure was ultimately transitioned to only spray coating due to its suitability for large‐scale production.^[^ ^86^ ^]^ In **Figure** **5**a, there is no significant difference between spin‐coated PEG and spray‐coated PEG, both of which achieve a maximum efficiency of ≈20% (with an average of 18%) across six solar devices, a notable improvement over the PSK1/PSK2 devices. Further analysis focuses on the speed of the spray nozzle, which influences the coverage and the amount of PEG solution applied. Speeds of 15, 20, and 25 mm s^−1^ were tested with the highest performance from the speed of 20 mm s^−1^ in Figure S14, Supporting Information. At 25 mm s^−1^, the PEG does not adequately cover the surface, rendering it ineffective, while at 15 mm s^−1^, excessive PEG acts as an insulating polymer, reducing performance.^[^ ^29^, ^66^, ^87^ ^]^

Introducing an interfacial modification between PSK1 and PSK2 whether by spin‐ or spray‐coating greatly improves device performance compared to the PSK1/PSK2 control, while the difference between spin and spray remains relatively minor. As shown in the external quantum efficiency (EQE) spectra in Figure S15, Supporting Information, the control device exhibits the lowest response, reflecting limited light absorption and charge collection, whereas both spin‐ and spray‐coated devices extend the response to ≈50%–60% across a broader spectral range, resulting in stronger photocurrent generation.^[^ ^22^ ^]^ The improvement is consistent with J–V measurements under 1000 lux LED as show in Figure 5b, where the control achieves only 14.3% PCE with low V_oc_ (0.79 V) and FF (0.514), while spin‐ and spray‐coated devices reach ≈19%–20% PCE with comparable V_oc_ (≈0.83 V), FF (≈0.60), and J_sc_ (≈124–127 μA cm^−2^). Stability tests using maximum power point tracking under continuous indoor illumination at 1000 lux, shown in Figure S16, Supporting Information, further validate the trend: both spin‐ and spray‐coated devices retain higher normalized PCE compared to the control, although their degradation behaviors are nearly identical. Whereas the control suffers from unstable and low power output, the modified devices maintain stable operation over time. The key improvement arises from the PEG interfacial modification itself, which reduces defect and enhances charge extraction,^[^ ^22^, ^66^ ^]^ while the choice between spin‐ and spray‐coating has only a marginal effect on efficiency and stability under real operating conditions.

Moreover, PEG coverage was confirmed through FTIR microscopy, as illustrated in Figure 5c. FTIR analysis can identify areas covered by PEG by irradiating them with infrared light at a wavenumber of 1094 cm^−1^, corresponding to the C—O stretching vibration between PEG and PSK. The transmittance measurements indicate no significant difference in PEG coverage between spin‐coating and spray‐coating.

Levelized cost of electricity (LCOE) is a key metric used to compare the long‐term cost‐effectiveness of electricity‐generating technologies. Based on the National Renewable Energy Laboratory (NREL) method,^[^ ^88^ ^]^ LCOE calculations consider three main input categories: performance, reliability, and finance. Performance includes device efficiency and energy yield without degradation. Reliability accounts for the degradation rate and service life, ensuring continued output over time. Financial inputs include the discount rate, which reflects the present value of future costs and energy production.^[^ ^89^, ^90^ ^]^

In this study, the LCOE was calculated under idealized conditions using the lowest reported market prices for materials from previous studies,^[^ ^91^ ^]^ a fixed efficiency of 20% for all structures, and standardized assumptions for balance of system cost, operation and maintenance cost, energy yield, system degradation rate, service life, and discount rate, as summarized in Table S7, Supporting Information.The LCOE values are generally determined under the standard sunlight condition; therefore, a modified LCOE for indoor light (m‐LCOE‐i) was introduced in this work with the calculation detail provided in Table S7, Supporting Information. These assumptions provide a benchmark for evaluating and comparing the cost potential of different perovskite solar cell stacks for indoor usage.

**Figure 6:**
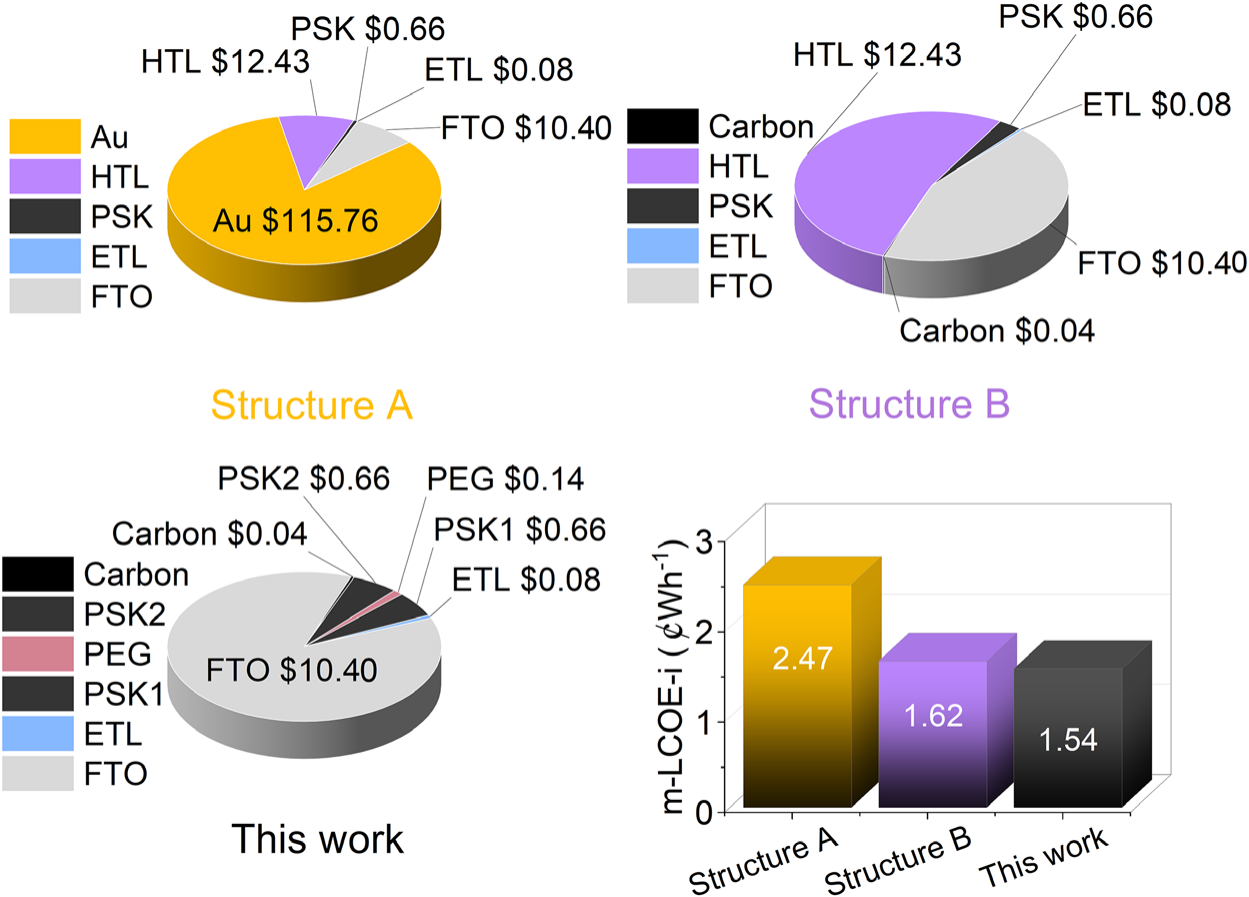
Cost comparison and modified LCOE analysis for indoor light (m‐LCOE‐i) of three PSC architectures: structure A (PSK/spiro‐OMeTAD/Au), structure B (PSK/spiro‐OMeTAD/carbon), and the proposed PSK1/PEG/PSK2/Carbon structure.

A comparison of material costs among three perovskite solar cell structures (**Figure** **6**) shows that this work achieves significantly lower costs and improves economic viability. While structures A and B rely on expensive components such as Spiro‐OMeTAD and/or gold (Au), resulting in total material costs per square meter of $139.33 and $23.62, respectively. Our proposed structure replaces expensive HTL with a PSK1/PEG/PSK2 layer along with the use of carbon, reducing the total material cost to $11.98, results in the lowest m‐LCOE‐i value of 1.54 ¢ Wh^−1^. This work demonstrates cost‐effectiveness via the uses of scalable spray coating, novel HTL replacement, and carbon electrode, viable for future large‐scale deployment in PSC production.

To enable a fair comparison with previous reports, we evaluated the cost using the actual efficiencies of each device, as earlier studies employed different active areas and fabrication methods that can significantly influence performance. Table S11, Supporting Information, shows the efficiency and cost scenarios, demonstrating that our work achieves the lowest m‐LCOE‐i. It is important to note that LCOE is typically assessed under large‐scale production conditions. While spin coating can deliver high efficiency in small‐area devices, it is not easily scalable for industrial manufacturing. In contrast, our spray‐coating method is compatible with large‐scale production,^[^ ^92^ ^]^ making the LCOE comparison more realistic and relevant for commercialization. Structure A (0.64 cm^2^, spin coating) exhibits a PCE of 30% with an m‐LCOE‐i of 1.91  ¢ Wh^−1^, Structure B (1 cm^2^, spin coating) has a PCE of 23% and an m‐LCOE‐i of 1.52 ¢ Wh^−1^, and our spray‐coated device (1 cm^2^) achieves a PCE of 21% with the lowest m‐LCOE‐i of 1.50 ¢ Wh^−1^. Although spray coating results in slightly lower PCE than spin coating, its scalability yields the most realistic and cost‐effective m‐LCOE‐i, highlighting its strong potential for large‐scale indoor photovoltaic applications.

## 3 Conclusions

This study advances the commercial viability of PSCs by integrating scalable manufacturing techniques like spray coating with cost‐effective materials. Traditional HTL was effectively replaced by dual perovskite absorber (PSK1/PSK2) with proper band alignment; the stack was enhanced by a PEG interlayer to effectively passivate interfacial defects in order to improve overall device performance. The devices demonstrate improved efficiency (up to 21%) and stability under various environmental conditions. m‐LCOE‐i calculation confirms a significant cost advantage over traditional Spiro‐based devices with either Au or carbon electrode. The practical use of the cost‐effective structure was illustrated by powering multiple IoT sensors over one month under a normal laboratory lighting environment at 342 lux.

## 4 Experimental Section

4.1

4.1.1

##### Chemicals and Materials

Lead (II) bromide (PbBr_2_, >98.0%) and lead (II) iodide (PbI_2_, 99.99%, trace metals basis) were purchased from Tokyo Chemical Industry (TCI). Formamidinium iodide (FAI, >99.99%), methylammonium bromide (MABr, >99.99%), formamidinium bromide (FABr, >99.99%), and TEC15 FTO glass substrats (thickness 2.2 mm) were purchased from Greatcell solar. Cesium iodide (CsI, 99.9%), methylammonium chloride (MACl, 98%), anhydrous N,N‐dimethylformamide (DMF, 99.8% v/v), anhydrous dimethyl sulfoxide (DMSO, 99% v/v), anhydrous ethanol (99.5% v/v), anhydrous chlorobenzene (99.8%), and tin(II) chloride dihydrate (SnCl_2_·2 H_2_O, 99.999%) were purchased from Sigma‐Aldrich. Tin(IV) oxide colloidal dispersion (15 wt% in water) was purchased from Alfa Aesar. Propan‐2‐ol (analytical reagent grade) was purchased from RCI Labscan. Alconox detergent powder was purchased from Alconox, while carbon paste (Jelcon CH‐8) was purchased from Jujo Chemical. Polyethylene glycol (PEG, with an average molecular weight of 6000) was purchased from Thermo Scientific Chemicals.

##### Device Fabrication

FTO glass substrates were cleaned by sonicating in an Alconox detergent solution for 30 min, followed by three rinses with water and one rinse with deionized water (DI water) then sonicated in isopropanol (IPA) for 30 min and dried using a nitrogen gun.

For the bilayer ETL, a 0.1 M solution of SnCl_2_·2H_2_O was deposited (100 μL) and spin‐coated onto the FTO substrate at 5000 rpm for 30 s, with an initial acceleration of 2500 rpm s^−1^. The films were then annealed at 180 °C for 1 h. For the second ETL layer, a 15 wt% SnO_2_ dispersion was diluted with DI water in a 1:2 ratio. A volume of 120 μL was deposited, spin‐coated under the same conditions, and annealed at 150 °C for 30 min.

The perovskite precursor solution for the first layer (PSK1) was prepared by dissolving CsI (30 mg), FAI (118 mg), MABr (14 mg), PbI_2_ (403 mg), and PbBr_2_ (138 mg) in a solvent mixture of DMF:DMSO (800:200 μL). Additionally, MACl (0.4 M, 5% by volume) was added to the solution.^[^ ^20^ ^]^ The mixture was stirred for 2 h at room temperature and filtered through a 0.22 μm PTFE syringe filter. For the second perovskite layer (PSK2), the same formulation was used with the addition of phenethylammonium iodide (PEAI) at a concentration of 1 mg mL^−1^.

For the passivation layer, polyethylene glycol (PEG, molecular weight 6000 g mol^−1^) was dissolved in chlorobenzene (CB) at a concentration of 1.5 mg mL^−1^. Complete dissolution was ensured to make the solution suitable for either spin coating or spraying.

The ETL‐coated substrates were treated with UV–ozone for 30 min and preheated at 130 °C for 30 s. A Badger 200 airbrush was mounted 20 cm above the substrate holder, which was attached to a motorized linear rail controlled by an Arduino microcontroller, allowing precise control of the scan speed. The perovskite precursor was delivered using industrial‐grade N_2_ gas at 30 psi, with a delivery rate of 25 μL s^−1^, and the scan speed at 10 mm s^‐1^. During deposition, the substrates were placed on a hot plate maintained at 130 °C. Following deposition, the films were annealed at 100 °C for 10 min to induce full crystallization.

For the control PSK1 film, PSK1 was sprayed twice followed by annealing. For the PSK1/PSK2 control film, PSK1 was sprayed once and annealed for 10 min, followed by spraying PSK2 once and annealing for another 10 min. For the optimized configuration, PSK1 was sprayed once and annealed for 10 min. The PEG passivation layer was then applied either by spraying or spin coating, using the same delivery rate as the perovskite precursor. The scan speed during spraying was set to 15, 20, or 25 mm s^−1^. Spin coating was performed at 4000 rpm for 30 s using 100 μL of the PEG solution. Finally, PSK2 was sprayed once and annealed for 10 min.

The carbon electrode was fabricated by casting Jelcon carbon paste preheated at 100 °C for 10 min prior to the experiment. An aluminum mask with a thickness of 120 μm and a 1 × 1 cm^−1^ center hole was used to define the active area. The carbon paste was then deposited onto the top perovskite layer using a doctor blade method and then the whole device was heated on a hot plate for 10 min at 100 °C, followed by baking in an oven at 70 °C for 30 min. The final carbon electrode thickness was ≈100 μm.

##### Characterizations

A field‐emission scanning electron microscope (FE‐SEM, JEOL JSM‐7610Plus) operating at an acceleration voltage of 7 kV was used to acquire surface and cross‐sectional images. AFM (Park NX‐10) was used with a noncontact probe (ACTA) with a resonance frequency of 300 kHz and a spring constant k = 37 N m^−1^ for producing noncontact phase and topographical images. A PANalytical Empyrean diffractometer was used for GI‐XRD. For wide‐angle scans, the scan step was 0.01°/step with time interval of 5.02 s/step, and the grazing angle was varied between 0.2°, 1°, 2°, 4°, 8°, and 10° with a 2*θ* range of 20°–40°. The same grazing angles were used for narrow‐angle scans, which had a resolution of 0.01°/step with time interval of 5.02 s/step and a 2*θ* range of 24°–29°. FTIR measurements were obtained by Nicolet iS50, Thermo Scientific, USA in the range between 400 and 4000 cm^−1^ with resolution of 4 cm^−1^ and a number of scans of 64. The coverage of the PEG layer was confirmed using optical microscopy (Olympus BX53MRF–S, 0E41082) and FTIR microscopy (Nicolet iN10 MX, transmittance mode). A D8 Discover Bruker X‐ray diffractometer (Cu Kα radiation, 2θ step size of 0.01°, acquisition period of 0.4 s per step) was used to evaluate the crystallinity of the film. A Horiba FluoroMax‐plus spectrophotometer was used to obtain steady‐state PL spectra (excitation at 500 nm, 0.3 s integration time, and 6 nm bandpass slit). PL lifetime measurements were performed from the substrate side and probed at 750 nm using the same technique when combined with a Horiba DeltaHub setup (excitation: 635 nm NanoLED, detection: 29.4 nm bandpass). The PAIOS platform was used to conduct electrochemical impedance spectroscopy (EIS) for the electrical and optical characterization of solar cells and LEDs. A Shimadzu UV‐2600 UV–Vis spectrophotometer was used to detect absorbance spectra across the 650–900 nm range. PL lifetime measurements were performed using the same system coupled with a Horiba DeltaHub setup (excitation: 635 nm NanoLED, detection: 29.4 nm bandpass), recorded from the substrate side and probed at 750 nm. EIS was performed using the PAIOS platform for electrical and optical characterization of solar cells and LEDs. Absorbance spectra were measured using a Shimadzu UV‐2600 UV–Vis spectrophotometer over a range of 650–900 nm. UPS was carried out at beamline 3.2Ua of the Synchrotron Light Research Institute, Thailand, with a photon energy of 60 eV. A Keithley 2400 source meter was used to measure the performance of indoor solar cells at a rate of 200 mV s^−1^ under 1000 lux (0.323 mW cm^−2^) illumination using a 6500 K Philips E27 LED (4 W) with a scan step of 0.05 V over a range of −0.1–1.2 V. A Si diode (Hamamatsu S1133) was used to calibrate the light intensity. An Enlitech QE‐R quantum efficiency analyser (DC mode with 1 mm^2^ beam diameter) was used to measure the EQE spectra.

## Supporting Information

Supporting Information is available from the Wiley Online Library or from the author.

## Conflict of Interest

The work is under patent filing process.

## Supporting information

Supplementary Material

## Data Availability

The data that support the findings of this study are available from the corresponding author upon reasonable request.
