# Supplementary material for: Economical Perovskite Solar Cell Enabled by Triple Cost‐Reduction Strategies
Source: Small Sci. 2026 Jan 19;6(1):e202500451. doi: 10.1002/smsc.202500451 (PMC12850025; doi:10.1002/smsc.202500451)
Supplement: Supplementary file 1 — Supplementary Material [file SMSC-6-e202500451-s001.pdf]

Supporting Information

Economical perovskite solar cell enabled by triple cost-reduction strategies

Kanokwan Choodam[1,2], Nattawut Kamjam[1,2], Noppawit Sukpan[1,2], Chaowaphat Seriwattanacha[1,2], Anuchytt Inna[1,2], KoKo Shin Thant[1,2], Ladda Srathongsian[1,2], Ratchadaporn Supruangnet[3], Hideki Nakajima[3], Anusit Kaewprajak[4], Pisist Kumnorkaew[4], Duangmanee Wongratanaphisan[5], Pipat Ruankham[5], Pasit Pakawatpanurut[6,7], Pongsakorn Kanjanaboos[1,2,7 *]

[1]School of Materials Science and Innovation, Faculty of Science, Mahidol University, Nakhon Pathom 73170, Thailand
[2]Center for Cooling and Energy-saving Materials, Faculty of Science, Mahidol University, Nakhon Pathom 73170, Thailand
[3]Synchrotron Light Research Institute, 111 University Avenue, Nakhon Ratchasima 30000, Thailand
[4]National Nanotechnology Center, National Science and Technology Development Agency, 114 Thailand Science Park, Phahonyothin Road, Khlong Luang, Pathum Thani 12120, Thailand
[5]Department of Physics and Materials Science, Faculty of Science, Chiang Mai University, Chiang Mai 50200, Thailand
[6]Department of Chemistry, Mahidol University, Bangkok 10400, Thailand
[7]Center of Excellence for Innovation in Chemistry, Ministry of Higher Education, Science, Research and Innovation, Bangkok 10400, Thailand

*Authors to whom correspondence should be addressed:*pongsakorn.kan@mahidol.edu

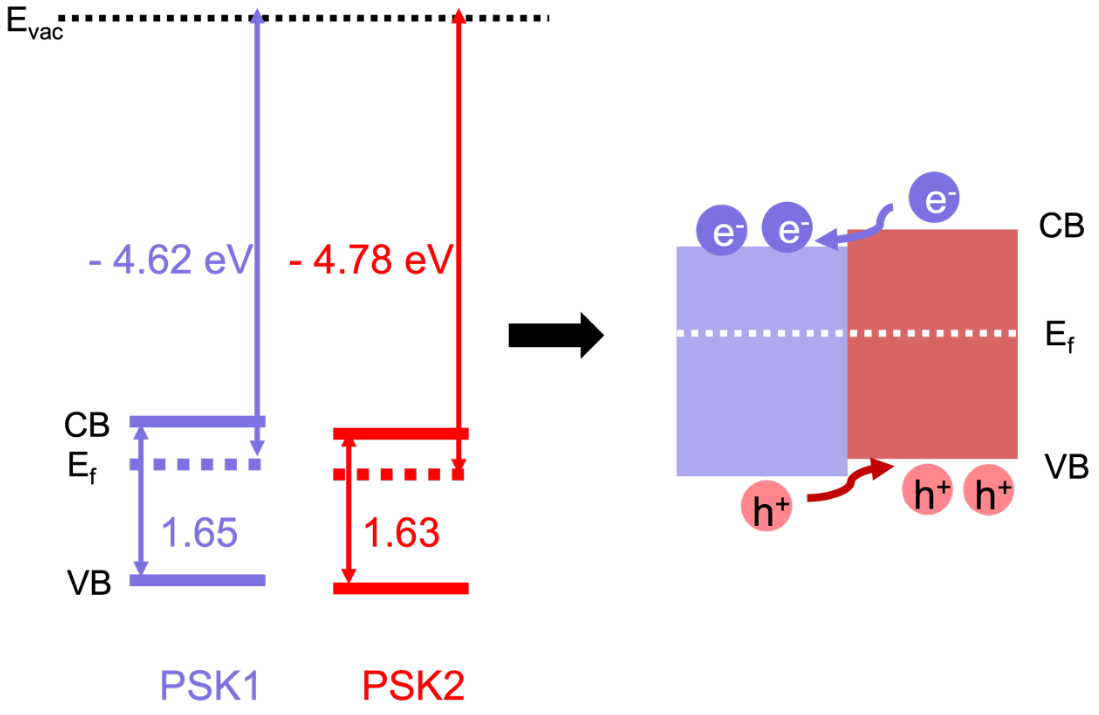

Figure S1. Schematic band alignment and charge transport mechanism of the dual-absorber structure (PSK1/PSK2).

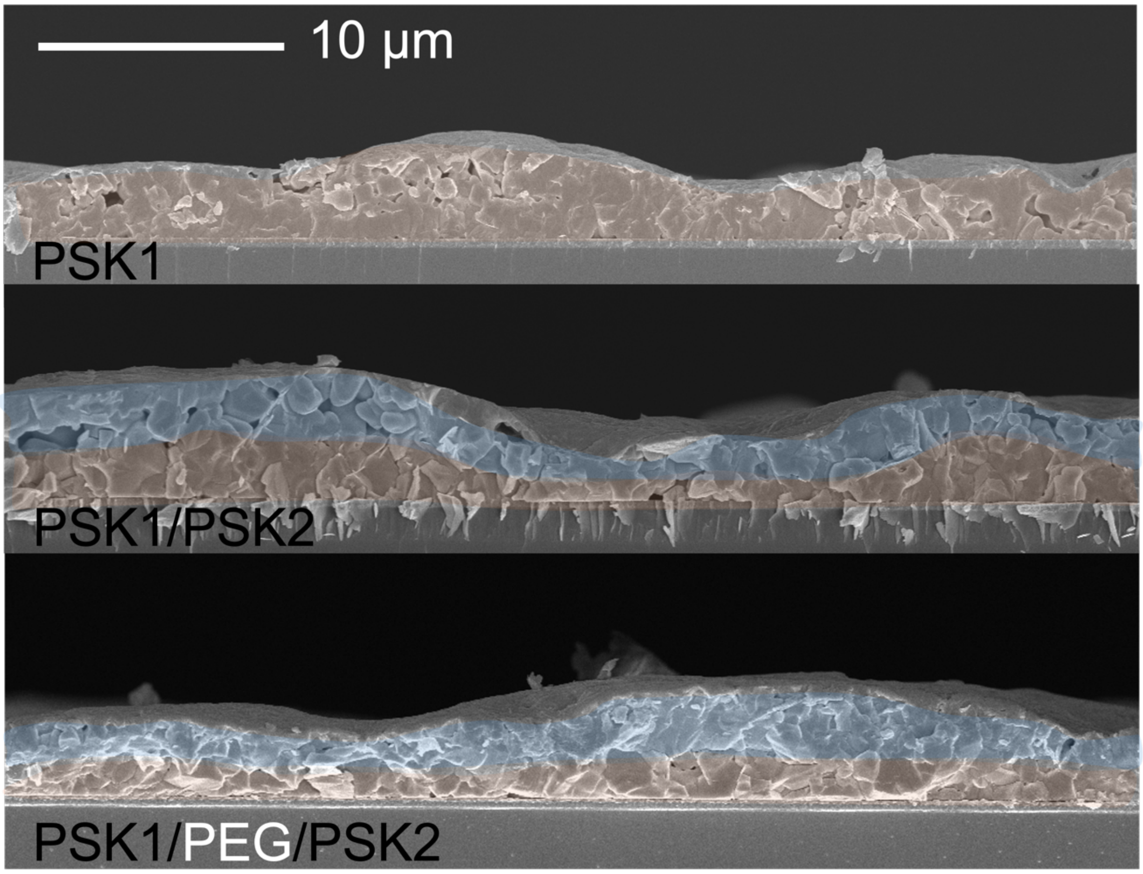

Figure S2. Cross-sectional SEM analysis of PSK1, PSK1/PSK2, and PSK1/PEG/PSK2 films.

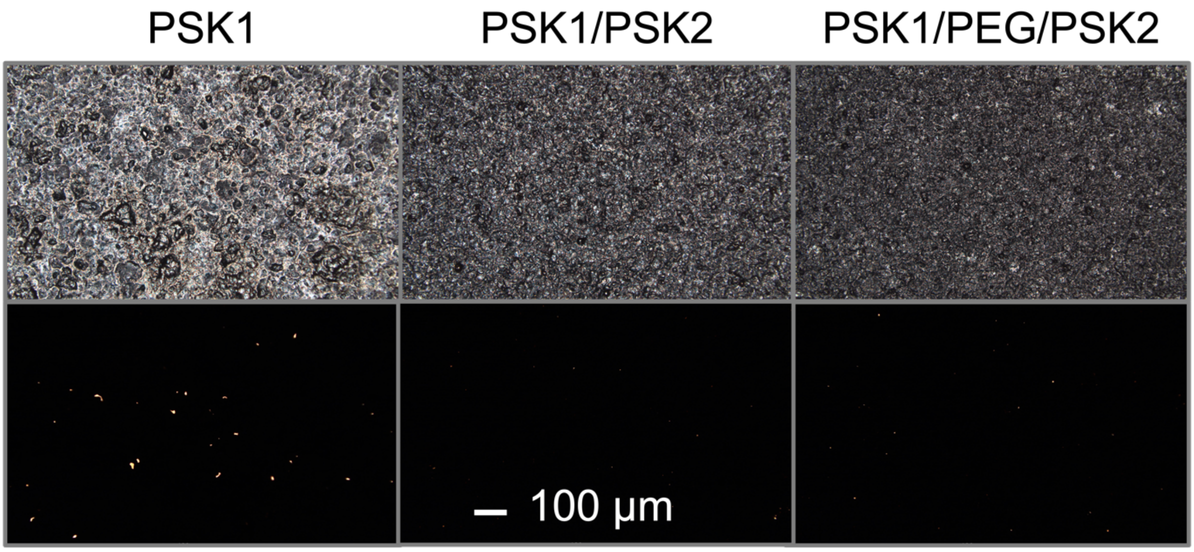

Figure S3. The optical microscope images were taken at 10x magnification with light from the top (top row) and bottom (bottom row) of PSK1, PSK1/PSK2, and PSK1/PEG/PSK2 films.

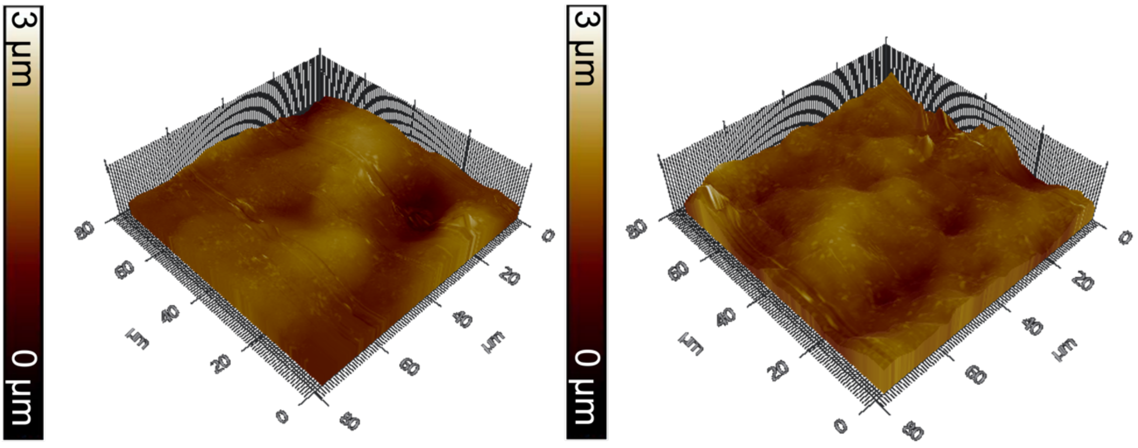

Figure S4. AFM analysis of surface roughness for glass/PSK1 and glass/PSK2 layers.

Table S1. Comparison of device configurations, fabrication methods, and performance metrics of representative perovskite solar cells with similar absorber structures.

Device configuration	Fabrication method	PCE	Key detail
FTO /SnO_2/$Cs_{0.05}FA_{0.81}MA_{0.14}PbI_{2.55}Br_{0.45}$/ $Cs_{0.10}FA_{0.81}MA_{0.14}PbI_{2.55}Br_{0.45}$/ Spiro-OMeTAD/Ag/Au	Spray coating	9.5% at 1 sun (0.049 cm^2)	Fast-drying spray coating (FDSC) was developed as a scalable method for fabricating multilayer perovskite absorbers, incorporating a graded energy band structure that enhances the built-in electric field and charge extraction. The process was further optimized to operate under high humidity without requiring post-annealing, offering a robust, low-cost, and scalable pathway for perovskite solar cell production.[1]
FTO/TiO_2/ $(PEA)_2(MA)_{n-1}Pb_nI_{n+1}Br_{2n}$ n∞/n50/ Spiro-OMeTAD/Au	Spray coating	3.32% at 1 sun (0.049 cm^2)	Introduced sequential spray deposition (SSD) to stack perovskite layers of different dimensionalities, enhancing both performance and stability. SSD functions under ambient humidity, offering a simple, cost-effective pathway for durable, large-scale perovskite photovoltaics.[2]
FTO/TiO_2/ CF_3-PEA)$_2FA_{0.85}MA_{0.15}Pb_2I_7$/ $FA_{0.85}MA_{0.15}PbI_3$/ Spiro-OMeTAD/Au	Spin coating	23.1% at 1 sun (0.090 cm^2)	A fluorinated 2D/3D perovskite heterojunction was successfully fabricated via interfacial ion exchange, where the 2D capping layer simultaneously passivates defects, enhances hydrophobicity, and forms a graded band structure for improved charge transport. Highlights the potential of fluorinated 2D interfaces for advancing efficient and durable perovskite photovoltaics.[3]
FTO/TiO_2/ $FAPbI_3$/ Octylammonium iodide/carbon	Spin coating	18.5% at 1 sun (0.1 cm^2)	Introducing a 2D perovskite passivation layer as an electron-blocking layer at the carbon electrode interface effectively suppresses recombination, leading to improved V_{OC}, FF, and stability.[4]
FTO/c-TiO_2/mTiO_2 $MAPbI_3$/$MAPbI_xBr_{3x}$/ carbon	Spin coating	16.2% at 1 sun (0.068 cm^2)	The $MAPbI_3$/$MAPbI_xBr_{3-x}$ perovskite stacking structure effectively improves the perovskite/carbon electrode interface, reducing interfacial recombination, enhancing charge extraction, and a promising strategy to optimize carbon electrode-based PSCs. [5]

Device configuration	Fabrication method	PCE	Key detail
FTO/Bilayer-SnO_2/PSK1/PEG/PSK2/carbon	Spray coating	21% at 1000 lux (1 cm^2)	The work demonstrates that dual perovskite with the PEG interlayer via ambient spray-coating and carbon electrode enable efficient, low-cost, and stable indoor PSCs. This architecture enhances charge transport, passivates defects, reduces material costs, and achieves a low m-LCOE-i, proving to be practical for powering IoT devices under typical indoor conditions.

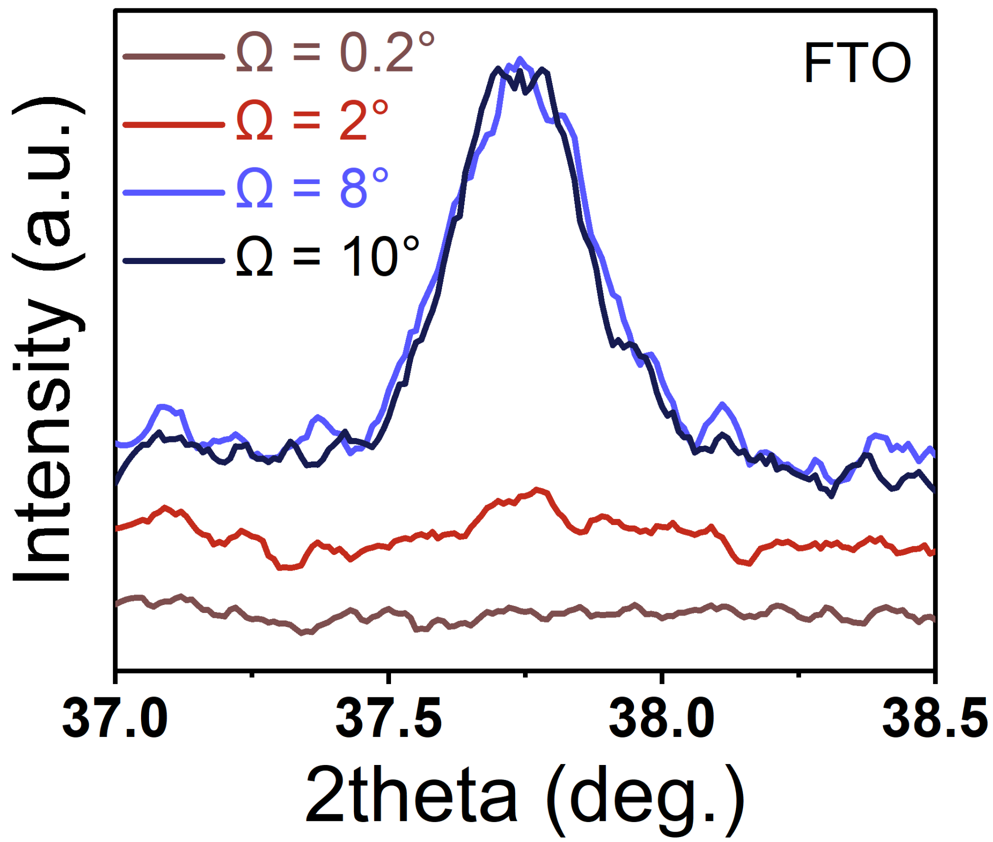

Figure S5. Grazing incidence XRD analysis of FTO peak at 0.2° - 10° grazing angles.

Table S2. The values of A_1, A_2, t_1, and t_2 for PSK1, PSK1/PSK2, and PSK1/PEG/PSK2.

**Parameter**	**PSK1**	**PSK1/PSK2**	**PSK1/PEG/PSK2**
A_1	10.96	18.45	9.49
t_1	56.79	50.93	47.62
A_2	89.04	81.55	90.51
t_2	988.26	648.53	999.50

Table S3. Comparison of different photovoltaic simulation tools.[6-8]

Simulator	Application	Strength	Limitation
SCAPS-1D (free and open source)	Si, GaAs, CdTe, CIS, CIGS, CZTS, Kesterite, Perovskites	SCAPS simulates optical and electrical properties, depicts energy band structures, and allows for up to seven semiconductor layers with varying materials and doping profiles. It uses drift-diffusion equations for carrier transport, combines light trapping and scattering models, and simulates I-V characteristics, quantum efficiency, spectral response, and fabrication procedures.	Longer simulation periods and can be ineffective with complex structures and materials compositions.
AMPS-1D (free basic plan or premium)	Si, GaAs,CdTe, CIGS, CZTS, Perovskites	Application for a one-dimensional simulator made for CIGS solar cells, which include architectures with homojunction, heterojunction, and multiple junctions. Crystalline, polycrystalline, and amorphous materials are all supported. It calculates characteristics for electrostatic potential and carrier current densities while simulating thin-film solar cells under a variety of situations, including temperature, light intensity, and materials attributes.	Tedious and time consuming due to the requirement of the large number of parameters and layers.
Gpvdm 1D/2D (free)	Si, GaAs,CdTe, CIGS, Perovskite, Organic	The software includes optical and electrical models to create precise simulations of solar cells, organic LEDs, OFETs, optical filters etc.	High computational demand and complexity cause steep learning curve and limit accessibility.

Simulator	Application	Strength	Limitation
Setfos 1D (commercial software)	Organic solar cells, OLEDs, Perovskite, QDs	Simulate light emission, absorption, scattering, and charge transport characteristics, ideal tool for the optimization of organic solar cell device structure.	Expensive, limited academic accessibility.
SilVACO TCAD 2D/3D (licensing options include perpetual, subscription and academic model)	Si, GaAs, a-Si, CdTe, CIGS, Multi-materials	Simulation of heterojunction and multijunction cells via methods like trap-limited carrier transport and the solution of transport, Poisson, and diffusion equations. Provide a thorough examination of electrical and optical properties.	Its high computational demands can be taxing, and its complexity may require extensive training.

In this work, we used the software developed by Marc Burgelman (Honorary Professor, University of Gent, Electronics and Information Systems, Campus Ardoyen, Technologiepark 914, B-9052 Gent-Zwijnaarde, Belgium). More details and downloads are available at: https://scaps.elis.ugent.be/SCAPSinstallatie.html.

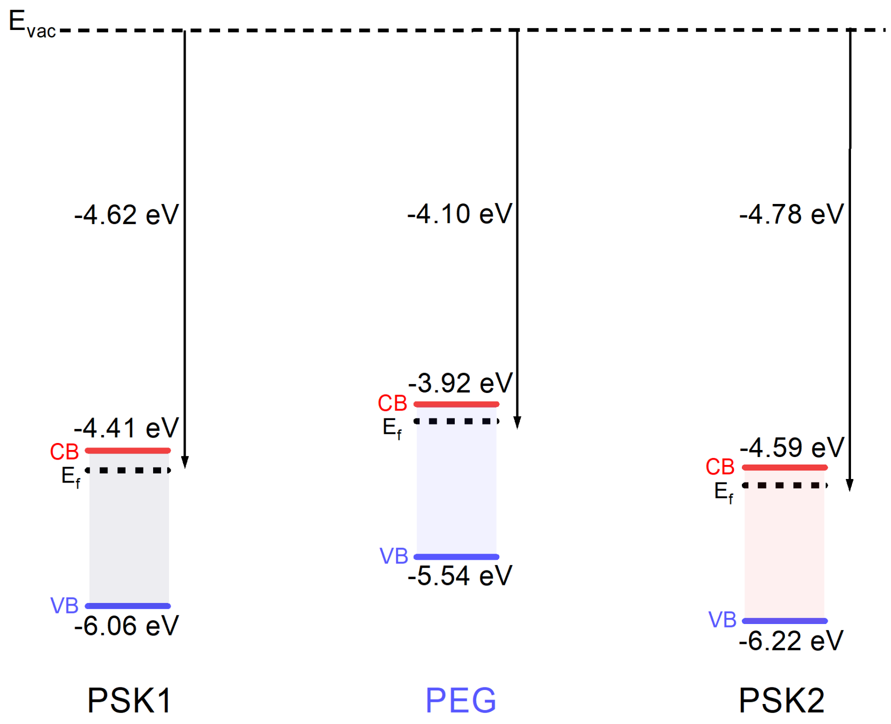

Figure S6. The energy band diagrams of PSK1, PSK1/PEG, and PSK2. The cut off energy of the perovskite layer was based from the previous research article [9].

Table S4. Electron affinity, thickness, band gap, and other parameters for SCAPs calculation.

Parameter	SnO_2	PSK1	PSK1/PEG	PSK2
Thickness (μm)	0.10	2.00	0.05	2.00
Bandgap (eV)	3.15	1.65	1.62	1.63
Electron affinity (eV)	4.52	4.41	3.92	4.59
Dielectric permittivity (relative)	9	30	3	10.00
CB effective density of states (cm^{-3})	2.20E+18	2.500E+20	2.800E+19	2.500E+20
VB effective density of states (cm^{-3})	1.80E+18	2.500E+20	1.000E+19	2.500E+20
Electron mobility (cm^2Vs^{-1})	7.56E-6	5.000E+1	1.000E-4	5.000E+1
Hole mobility (cm^2Vs^{-1})	1.00E-1	5.000E+1	1.000E-4	5.000E+1
Shallow uniform donor/ acceptor density density (cm^{-3})	1.00E+21/-	-/1.00 E+17	-/1.00 E+18	-/1.00 E+17

The value of other parameter from literature [10-13]

Table S5. The conduction band offset (CBO)[12, 14] for the PSK1, PSK1/PSK2, and PSK1/PEG/PSK2 structures.

CBO (PSK1/PEG/PSK2)	$\chi_{PSK1/PEG} - \chi_{PSK1}$	+ 0.49 eV
CBO (PSK2/PSK1)	$\chi_{PSK2} - \chi_{PSK1}$	- 0.18 eV
CBO (PSK1/PSK1)	$\chi_{PSK1} - \chi_{PSK1}$	0 eV

where CBO is calculated by subtracting χ or the affinity of materials, which are conduction band values acquired from UPS and UV-vis experiments.

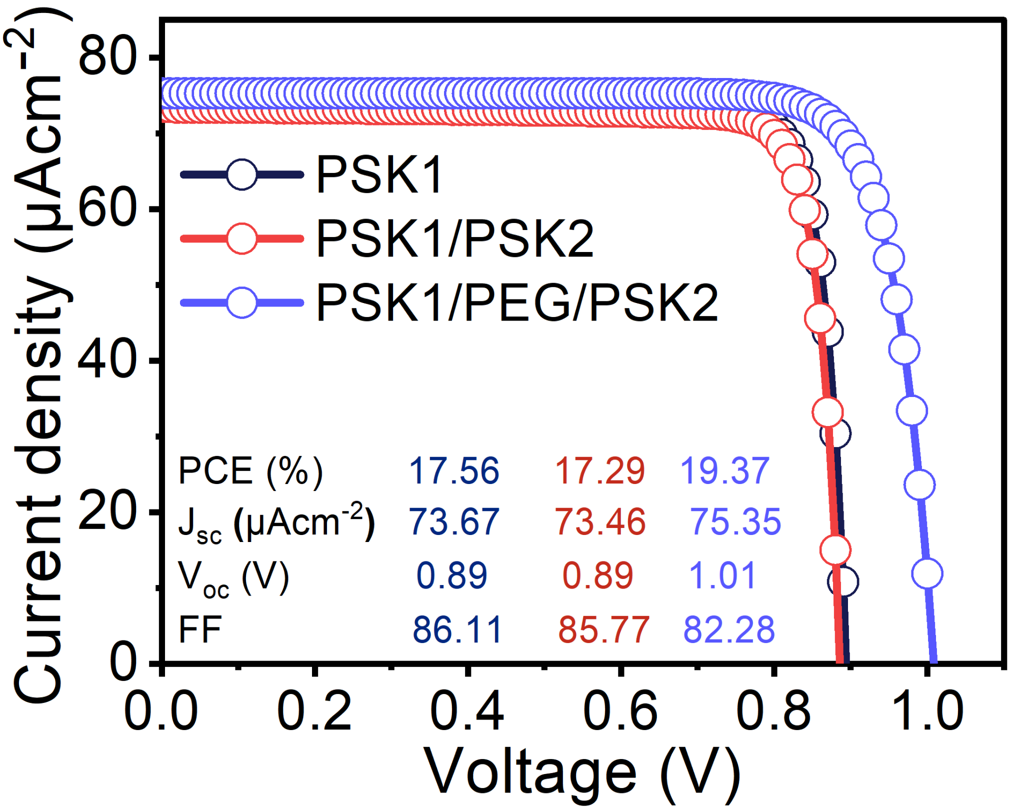

Figure S7. Simulation results for PSK1, PSK1/PSK2 and PSK1/PEG/PSK2 structures.

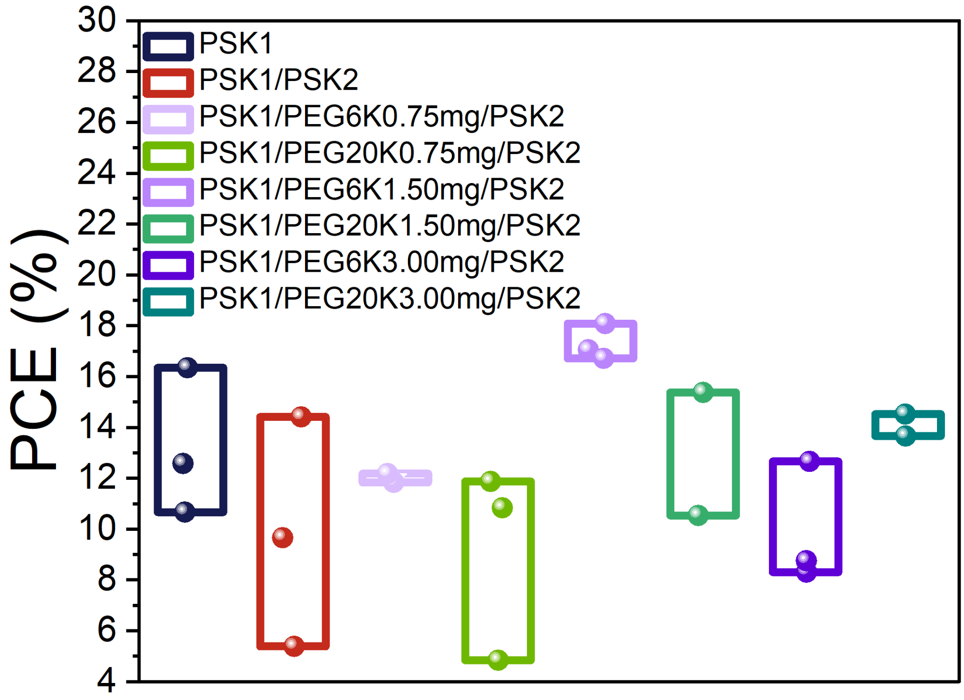

Figure S8. Device performances from varied concentrations and molecular weights of PEG.

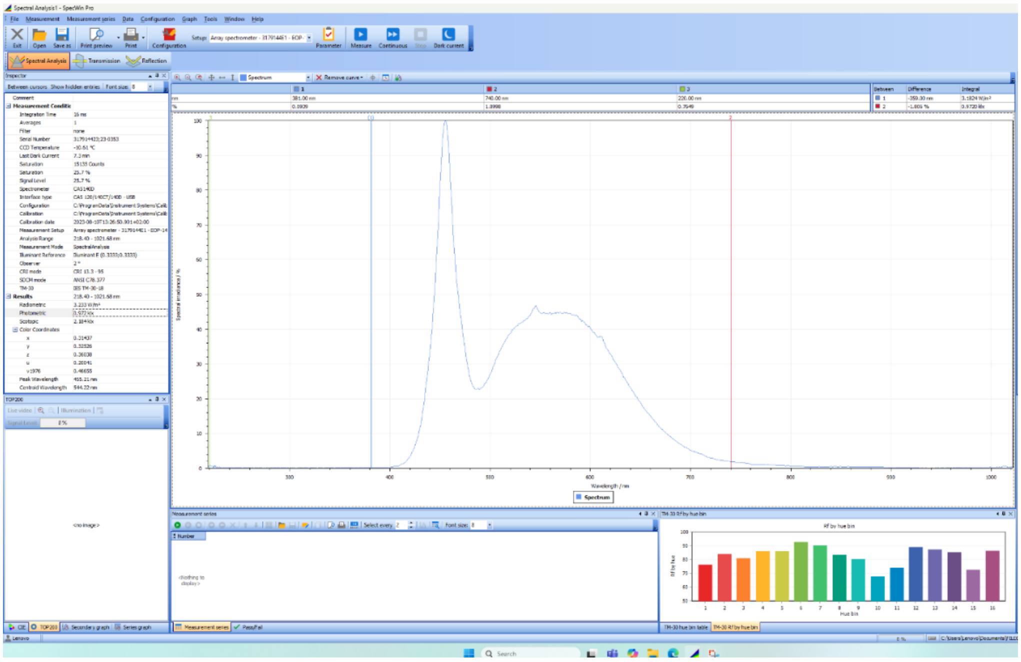

Figure S9. The indoor light source spectrum of the testing box (6500 K LED) was directly measured using a spectroradiometer with a certified EOP-146 detector.

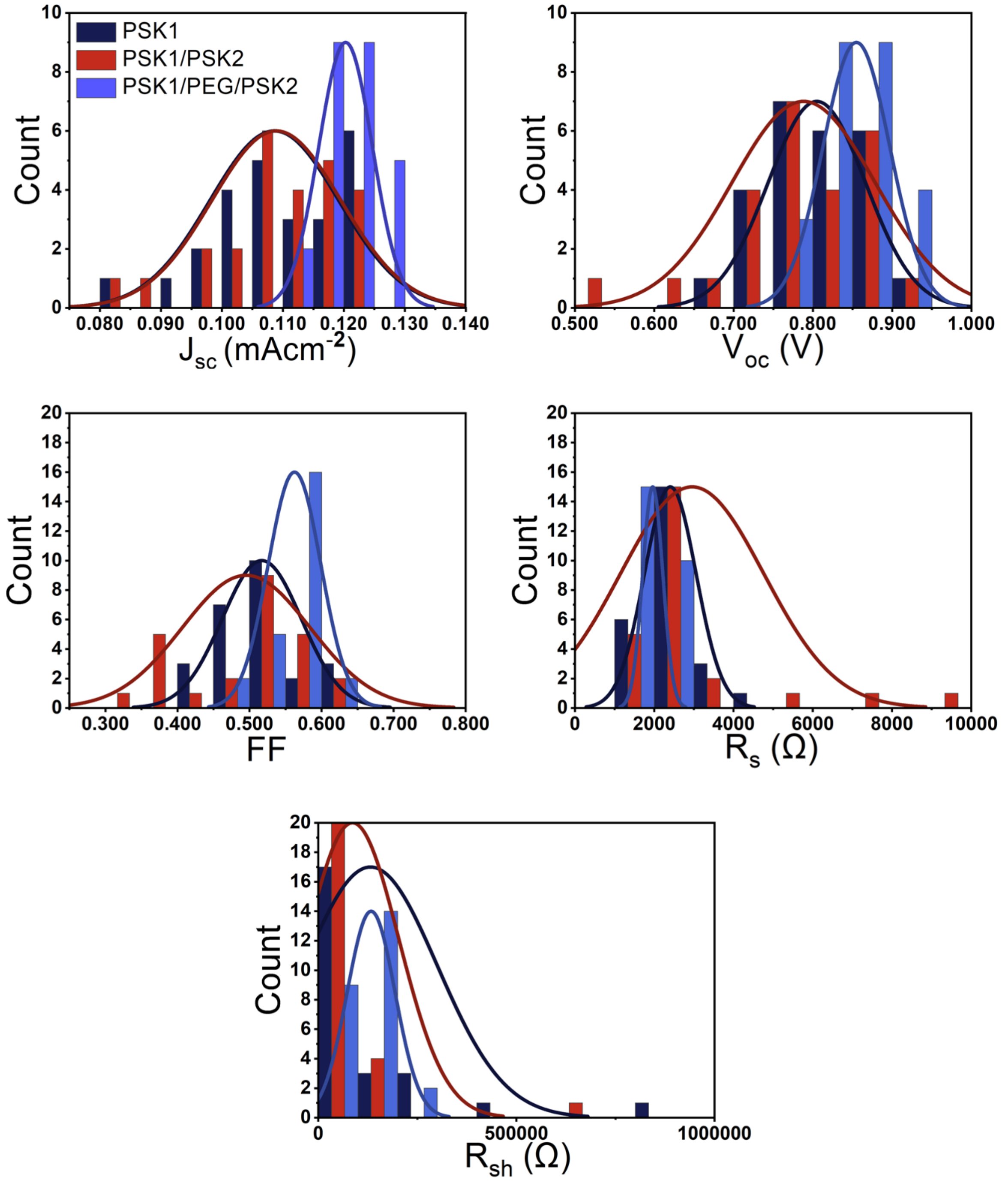

Figure S10. J_{sc}, V_{oc}, FF, R_s, and R_{sh} of PSK1, PSK1/PSK2, and PSK1/PEG/PSK2 devices.

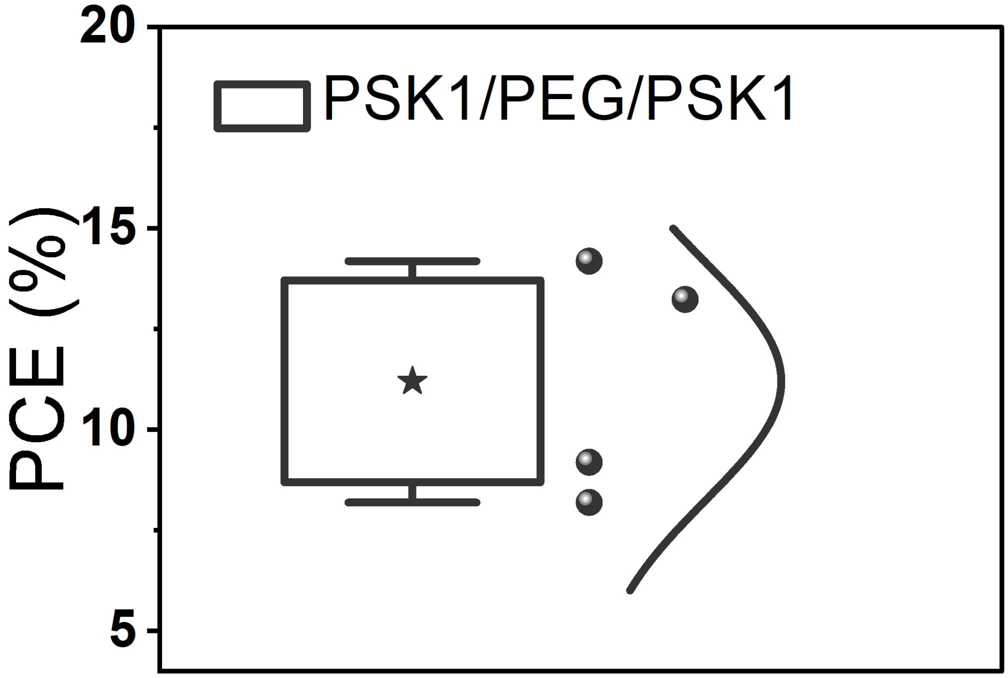

Figure S11. Device performance of PSK1/PEG/PSK1 structure.

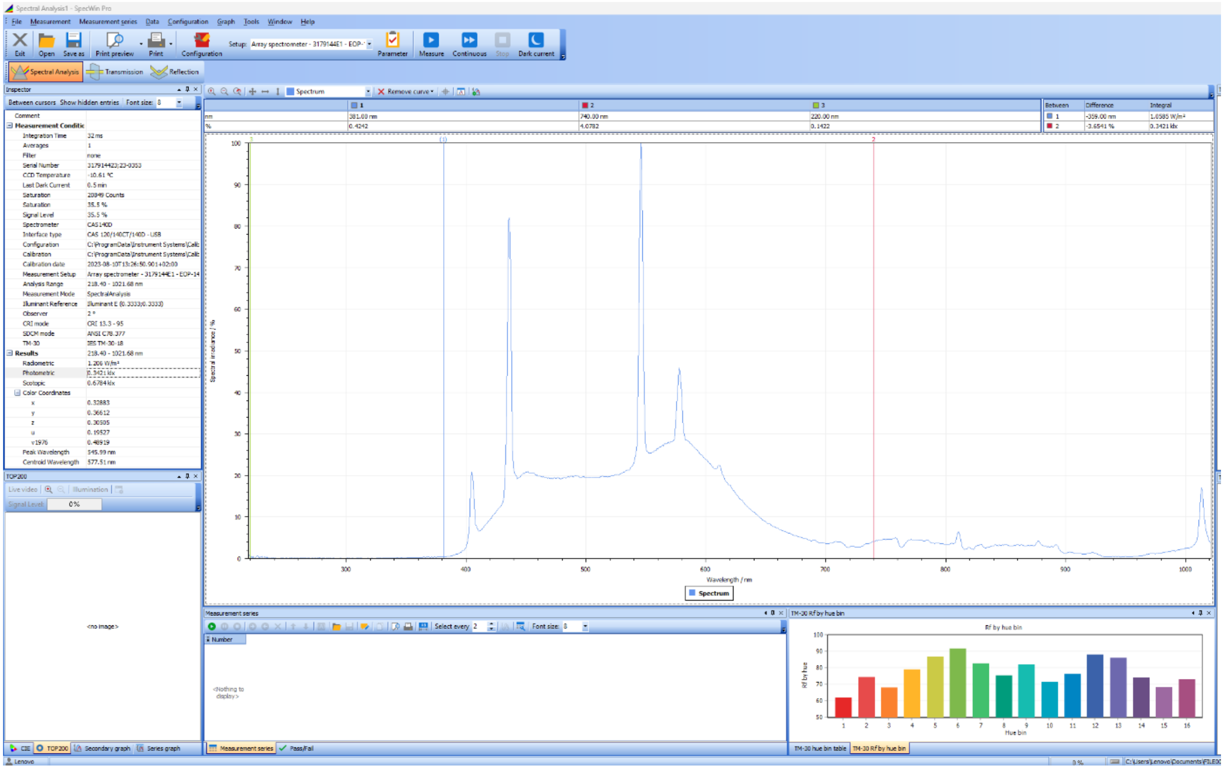

Figure S12. Indoor light source spectrum used for testing the performance of a PSK1/PEG/PSK2 device for IoT.

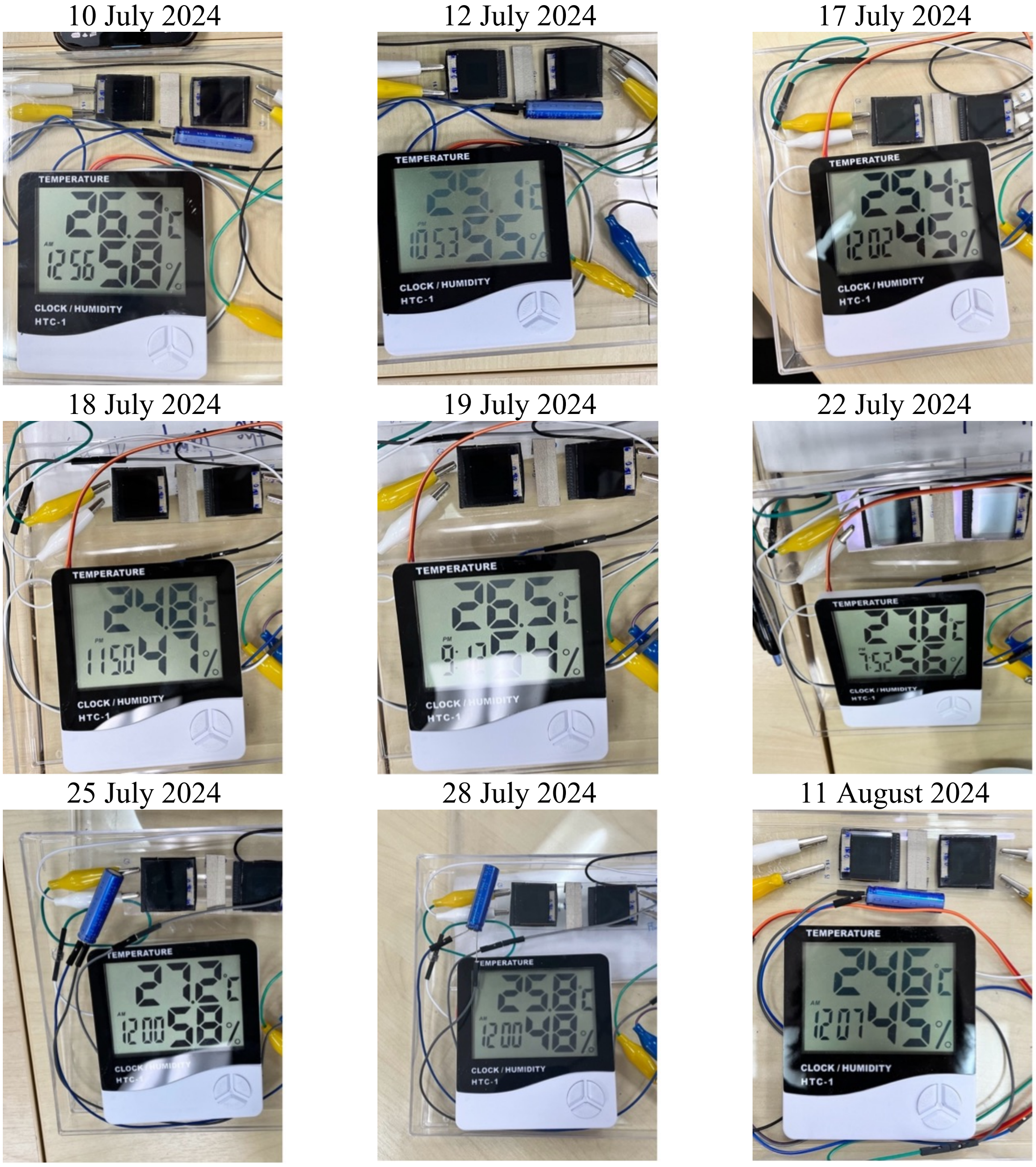

Figure S13. PSK1/PEG/PSK2 connected devices for powering low-power internet of things (IoTs) under normal indoor light ambience at 50-85%RH and on/off laboratory light cycle (~16 h light on and ~8 h light off).

Table S6. Photovoltaic performance of the device and its usage potential

Parameter	Value
PCE	13.816 %
J_{sc}	0.062 $mAcm^{-2}$
V_{oc}	1.770 V
FF	0.408
R_{sh}	70306.80 Ω
R_s	6291.22 Ω
Active area	2 cm^2
Power generation (solar cell)	0.0896 mW
Power consumption (sensor)	0.0275 mW

$P_{solar\ cell}$ = $(V_{oc})(I_{sc})(FF)$; $I_{sc} = (J_{sc})(Area) = (0.000062)(2)$ A
= (1.77)(0.000124)(0.408)
= 0.0896 mW

P_{sensor} = IV (the values from the actual measurement)
= (0.0183)(1.5)
= 0.0275 mW

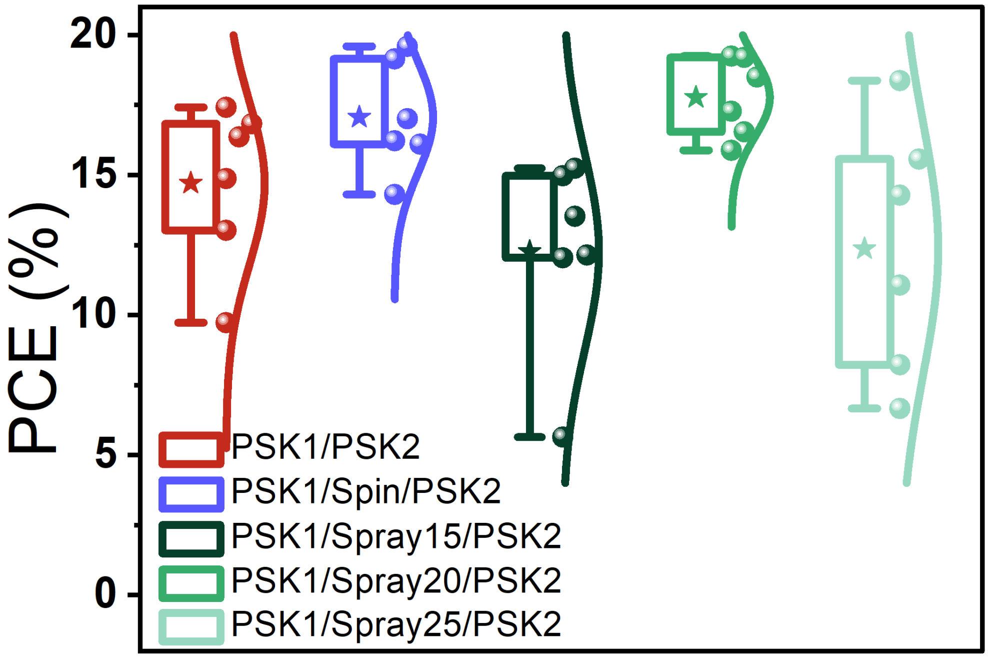

Figure S14. Optimization of spray nozzle speeds (15, 20, and 25 mms^{-1}) for PEG layer deposition and corresponding PSC performances.

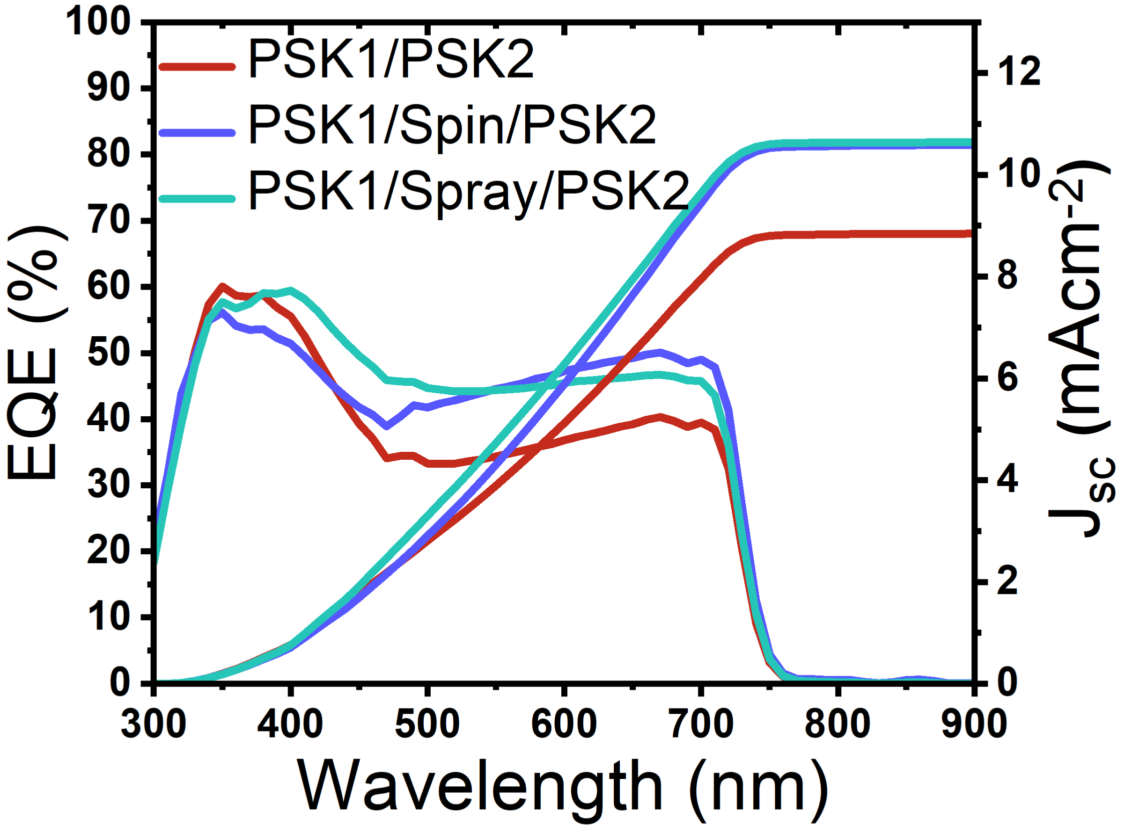

Figure S15. The EQE spectra for PSK1/PSK2, PSK1/spin-coated PEG/PSK2, and PSK1/spray-coated PEG/PSK2 structures.

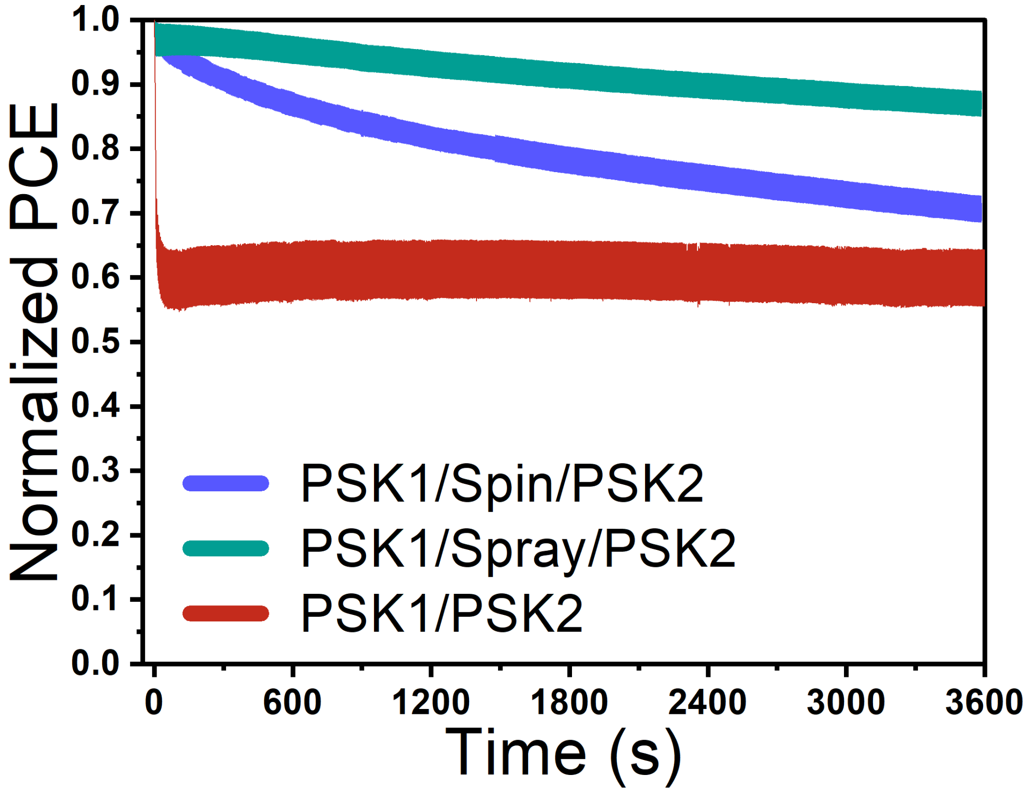

Figure S16. Stability tests using maximum power point tracking (MPPT) under continuous indoor illumination at 1000 lux.

Table S7. Assumed parameters for idealized LCOE estimation including baseline, performance, reliability, and finance.

Category	Parameter	Value
Baseline	Cell cost ($\$m^{-2}$)	Calculated from the lowest prices of materials listed [15]
	O&M Cost ($\$kW^{-1}year^{-1}$)	18.71[15, 16]
	BOS cost, power-scaling ($\$W^{-1}$)	0.36[15, 16]
	BOS cost, area-scaling ($\$m^{-2}$)	103.59[15, 16]
Performance	Efficiency (%)	20% for all structure
	Energy yield ($kWhkW^{-1}$)	1848[15, 16]
Reliability	System degradation rate ($\%year^{-1}$)	0.648[15, 16]
	Service life (year)	25[15, 16]
Financial	Discount rate (%)	5[15, 16]

We calculated the LCOE values based the NREL website data above via the NREL calculator tool[16], yielding 7.98, 5.24, and 4.97 ¢kWh^{-1} under 100 $mWcm^{-2}$ for structure A, structure B, and this work, respectively. However, typical indoor lighting at 1000 lux provides only 0.323 $mWcm^{-2}$, a difference of approximately 309.6 times (100/0.323 = 309.6). Therefore, the modified LCOE for indoor light (m-LCOE-i) can be calculated by multiplying LCOE values for one sun by this factor, resulting in 2.47, 1.62, and 1.54 ¢Wh^{-1} under 1000 lux for structure A, structure B, and this work, respectively.

Table S8. Calculated cell cost of structure A based on the lowest reported materials prices.

Structure A	
**Material**	**Cost ($)**
Glass-FTO	10.4
SnO_2	0.00425
EtOH	0.08
CsI	0.0024
MABr	0.00164
FAI	0.5232
$PbBr_2$	0.011
PbI_2	0.0436
DMF	0.005
DMSO	0.07
Spiro+Li+TBP	12.433
CB	0.001
Au	115.758
**Sum**	139.33

Table S9. Calculated cell cost of structure B based on the lowest reported materials prices.

Structure B	
**Material**	**Cost ($)**
Glass-FTO	10.4
SnO_2	0.00425
CsI	0.0024
EtOH	0.08
MABr	0.00164
FAI	0.5232
$PbBr_2$	0.011
PbI_2	0.0436
DMF	0.005
DMSO	0.07
Spiro+Li+TBP	12.433
CB	0.001
Carbon	0.0436
**Sum**	23.61869

Table S10. Calculated cell cost of this work based on the lowest reported materials prices.

**This work**	
**Material**	**Cost ($)**
Glass-FTO	10.4
SnO_2	0.00425
EtOH	0.08
CsI	0.0024
MABr	0.00164
FAI	0.5232
$PbBr_2$	0.011
PbI_2	0.0436
DMF	0.005
DMSO	0.07
PEG	0.1392
CsI	0.0024
MABr	0.00164
FAI	0.5232
$PbBr_2$	0.011
PbI_2	0.0436
DMF	0.005
DMSO	0.07
Carbon	0.0436
**Sum**	11.98

Table S11. Modified levelized cost of electricity for indoor light (m-LCOE-i) comparison for three perovskite solar cell architectures.

**Structure**	**PSK Method**	**PCE at 1000 lux (active area)**	**m-LCOE-i (¢Wh^{-1})**	**Ref**
Structure A	Spin coating	30% (0.64 cm^2)	1.91	[17]
Structure B	Spin coating	23% (1 cm^2)	1.52	[18]
This work	Spray coating	21% (1 cm^2)	1.50	

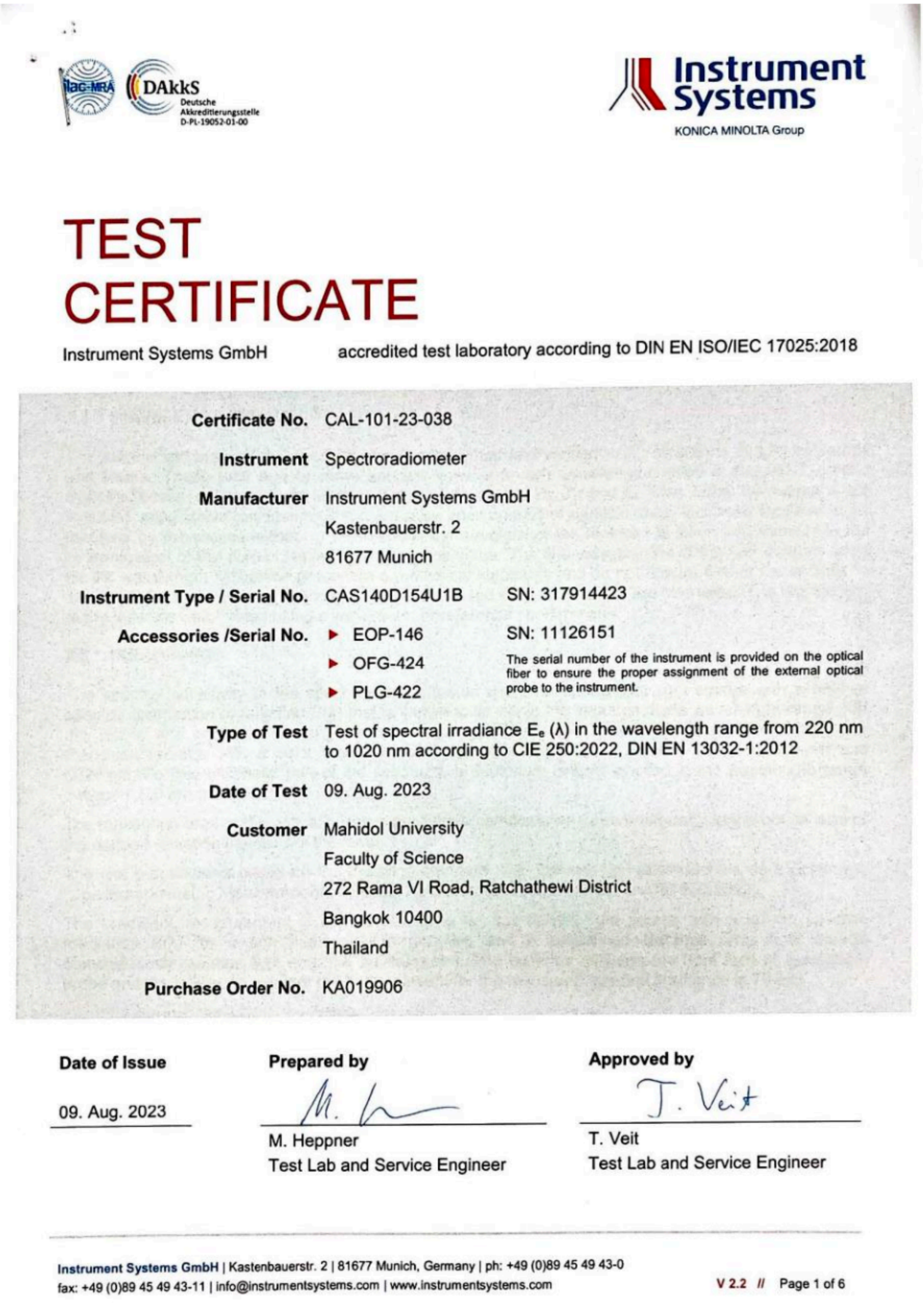

DAkkS Deutsche Akkreditierungsstelle D-PL-19052-01-00

Instrument Systems
KONICA MINOLTA Group

TEST CERTIFICATE

Instrument Systems GmbH accredited test laboratory according to DIN EN ISO/IEC 17025:2018

**Certificate No.**	CAL-101-23-038
**Instrument**	Spectroradiometer
**Manufacturer**	Instrument Systems GmbH<br>Kastenbauerstr. 2<br>81677 Munich
**Instrument Type / Serial No.**	CAS140D154U1B SN: 317914423
**Accessories /Serial No.**	▸ EOP-146 SN: 11126151<br>▸ OFG-424 The serial number of the instrument is provided on the optical fiber to ensure the proper assignment of the external optical probe to the instrument.<br>▸ PLG-422
**Type of Test**	Test of spectral irradiance E_e (λ) in the wavelength range from 220 nm to 1020 nm according to CIE 250:2022, DIN EN 13032-1:2012
**Date of Test**	09. Aug. 2023
**Customer**	Mahidol University<br>Faculty of Science<br>272 Rama VI Road, Ratchathewi District<br>Bangkok 10400<br>Thailand
**Purchase Order No.**	KA019906

Date of Issue	Prepared by	Approved by
09. Aug. 2023	M. Heppner<br>Test Lab and Service Engineer	T. Veit<br>Test Lab and Service Engineer

Instrument Systems GmbH | Kastenbauerstr. 2 | 81677 Munich, Germany | ph: +49 (0)89 45 49 43-0
fax: +49 (0)89 45 49 43-11 | info@instrumentsystems.com | www.instrumentsystems.com

V 2.2 // Page 1 of 6

Figure S17. Test certificate of the spectroradiometer**.**

Raw and simulation files for this work can be found via this link:

https://drive.google.com/drive/folders/1dzNA90RVy2V1rhCSOd0XBrfSSAef_Smn?usp=drive_link

Reference:

1. W. Tuchinda, K. Amratisha, A. Naikaew, P. Pansa-Ngat, L. Srathongsian, W. Wattanathana, K. K. S. Thant, R. Supruangnet, H. Nakajima, P. Ruankham, *Sol. Energy* 2022, **244**, 65.
2. K. Amratisha, J. Ponchai, P. Kaewurai, P. Pansa-Ngat, K. Pinsuwan, P. Kumnorkaew, P. Ruankham, P. Kanjanaboos, *Opt. Mater. Express* 2020, **10** (7), 1497.
3. Y. Cai, J. Wen, Z. Liu, F. Qian, C. Duan, K. He, W. Zhao, S. Zhan, S. Yang, J. Cui, *J. Energy Chem.* 2022, **65**, 480.
4. S. Zouhair, S. M. Yoo, D. Bogachuk, J. P. Herterich, J. Lim, H. Kanda, B. Son, H. J. Yun, U. Würfel, A. Chahboun, *Adv. Energy Mater.* 2022, **12** (21), 2200837.
5. D. Li, L. Chao, C. Chen, X. Ran, Y. Wang, T. Niu, S. Lv, H. Wu, Y. Xia, C. Ran, *Nano Lett.* 2020, **20** (8), 5799.
6. A. Kowsar, M. Billah, S. Dey, S. C. Debnath, S. Yeakin, S. F. U. Farhad, *Proc. 2nd Int. Conf. Innov. Eng. Technol. (ICIET)*, 23–24 Dec. 2019, pp. 1–6. DOI: 10.1109/ICIET48527.2019.9290675.
7. M. D. Jones, J. A. Dawson, S. Campbell, V. Barrioz, L. D. Whalley, Y. Qu, *Front. Chem.* 2022, **10**, 920676.
8. L. Salgado-Conrado, C. Álvarez-Macías, B. Reyes-Durán, *Mater.* 2024, **17** (21), 5213.
9. Y. Luan, X. Yi, P. Mao, Y. Wei, J. Zhuang, N. Chen, T. Lin, C. Li, J. Wang, *iScience* 2019, **16**, 433.
10. M. Mehrabian, E. N. Afshar, O. Akhavan, *Mater. Sci. Eng. B* 2023, **287**, 116146.
11. S. R. Hosseini, M. Bahramgour, N. Delibas, A. Niaei, *J. Optoelectron. Nanostruct.* 2022, **7** (2), 37.
12. T. Gou, Y. Li, Z. Lv, M. Zhao, J. Dai, F. Cao, *Micro Nano Struct.* 2024, **189**, 207819.
13. M. Meskini, S. Asgharizadeh, *Sci. Rep.* 2024, **14** (1), 5723.
14. M. A. Nalianya, C. Awino, H. Barasa, V. Odari, F. Gaitho, B. Omogo, M. Mageto, *Optik* 2021, **248**, 168060.
15. K. K. S. Shin Thant, C. Seriwattanachai, T. Jittham, N. Thamangraksat, P. Sakata, P. Kanjanaboos, *Adv. Energy Mater.* 2025, **15** (5), 2403088.
16. S. J. Andrews, B. S. Miller, M. G. Deceglie, K. A. Horowitz, T. J. Silverman, *Comparative Photovoltaic Levelized Cost of Energy Calculator*, 2021. [Online]. Available: https://www.nrel.gov/pv/lcoe-calculator/.
17. C. Teixeira, P. Spinelli, L. A. Castriotta, D. Müller, S. Öz, L. Andrade, A. Mendes, A. D. Carlo, U. Würfel, K. Wojciechowski, *Adv. Funct. Mater.* 2022, **32** (40), 2206761.
18. A. Naikaew, S. Burimart, L. Srathongsian, C. Seriwattanachai, P. Sakata, K. Choodam, K. Khotmungkhun, W. Kanlayakan, P. Pansa-Ngat, K. K. S. Thant, *Sol. RRL* 2025, **9** (8), 2400910.
